# Reciprocal interplay between OTULIN–LUBAC determines genotoxic and inflammatory NF-κB signal responses

**DOI:** 10.1073/pnas.2123097119

**Published:** 2022-08-08

**Authors:** Mingqi Li, Ling Li, Sarah Asemota, David Kakhniashvili, Ramesh Narayanan, Xusheng Wang, Francesca-Fang Liao

**Affiliations:** ^a^Department of Pharmacology, Addiction Science, and Toxicology, College of Medicine, University of Tennessee Health Science Center, Memphis, TN 38163;; ^b^Department of Biology, University of North Dakota, Grand Forks, ND 58202;; ^c^Department of Medicine, College of Medicine, University of Tennessee Health Science Center, Memphis, TN 38103;; ^d^Proteomics & Metabolomics Core Facility, Office of Research, College of Medicine, University of Tennessee Health Science Center, Memphis, TN 38163

**Keywords:** NF-κB, OTULIN, deubiquitinases, LUBAC, inflammation

## Abstract

Targeting nuclear factor-kappa B (NF-κB) represents a highly viable strategy against chemoresistance in cancers as well as cell death. Ubiquitination, including linear ubiquitination mediated by the linear ubiquitin chain assembly complex (LUBAC), is emerging as a crucial mechanism of overactivated NF-κB signaling. Ovarian tumor family deubiquitinase OTULIN is the only linear linkage–specific deubiquitinase; however, the molecular mechanisms of how it counteracts LUBAC-mediated NF-κB activation have been largely unknown. Here, we identify Lys64/66 of OTULIN for linear ubiquitination facilitated in a LUBAC-dependent manner as a necessary event required for OTULIN–LUBAC interaction under unstressed conditions, which becomes deubiquitinated by OTULIN itself in response to genotoxic stress. Furthermore, this self-deubiquitination of OTULIN occurs intermolecularly, mediated by OTULIN dimerization, resulting in the subsequent dissociation of OTULIN from the LUBAC complex and NF-κB overactivation. Oxidative stress induces OTULIN dimerization via cysteine-mediated covalent disulfide bonds. Our study reveals that the status of the physical interaction between OTULIN and LUBAC is a crucial determining factor for the genotoxic NF-κB signaling, as measured by cell survival and proliferation, while OTULIN loss of function resulting from its dimerization and deubiquitination leads to a dissociation of OTULIN from the LUBAC complex. Of note, similar molecular mechanisms apply to the inflammatory NF-κB signaling in response to tumor necrosis factor α. Hence, a fuller understanding of the detailed molecular mechanisms underlying the disruption of the OTULIN–LUBAC interaction will be instrumental for developing future therapeutic strategies against cancer chemoresistance and necroptotic processes pertinent to numerous human diseases.

## Significance

Deubiquitinases have emerged as a new class of modulators governing nuclear factor-kappa B (NF-κB) signaling. Ovarian tumor family deubiquitinase OTULIN inhibits NF-κB activation via counteracting the linear ubiquitin chain assembly complex (LUBAC). Clinically, OTULIN loss of function leads to OTULIN-related autoinflammatory syndrome. Here, we demonstrate that OTULIN loss of function leads to chemoresistance in experimental cancer models. Moreover, we discover molecular events under normal and genotoxic/inflammatory conditions involving LUBAC-dependent linear ubiquitination and oxidative stress–mediated dimerization of OTULIN through disulfide bonds. These events are strongly supported in clinical specimens of chemoresistant breast tissue. Given the central role of NF-κB–mediated hyperinflammation upon current pandemic, strategies focusing on stabilizing the OTULIN–LUBAC interaction may provide options in future drug development.

The nuclear factor-kappa B (NF-κB) family is arguably one of the most important transcription factors activated in response to various stimuli, including proinflammatory cytokines and environmental stresses such as genotoxic stress. NF-κB signaling regulates diverse processes such as cell survival/death, inflammation, immunity, and cancer (1–3). Given its role in such crucial processes, NF-κB signaling must be subjected to strict spatiotemporal control. Dysregulation of NF-κB signaling can be debilitating and often leads to lethal inflammation, such as in some types of cancer as well as severe cases of severe acute respiratory syndrome coronavirus 2 (SARS-CoV-2) (1, 4).

Over the past three decades, the key components of the signaling pathways leading to the activation of NF-κB have mostly been identified, including the prominent roles of protein phosphorylation (3) and ubiquitination (5, 6). Moreover, our knowledge regarding the negative feedback mechanisms targeting different components of the NF-κB signaling cascade has been rapidly expanding, from the initial direct regulation of the critical inhibitor of κB (IκB) proteins (e.g., small ubiquitin-like modifier/SUMO modification of IκBα) (7) to receptor-mediated signaling complexes (8). A complete understanding of the homeostatic feedback mechanisms that counteract and fine-tune inducible activation of NF-κB is of paramount importance since its dysregulation directly contributes to human diseases.

Ubiquitination is a critical process in NF-κB activation and inhibition (9). Of note, many components of the ubiquitination process during the activation of NF-κB have been illustrated, while several molecules in the deubiquitination process have been identified as negatively modulating NF-κB activity. In unstimulated cells, NF-κB is sequestered in the cytoplasm via binding to IκBα, which is phosphorylated by the IκB kinase (IKK) complex upon activation. The IKK family is composed of IKKα, IKKβ, and the NF-κB essential modulator (NEMO/IKKγ). Genotoxic stress–induced NEMO sumoylation and ubiquitination are required for the NF-κB activation process (10). Under tumor necrosis factor alpha (TNFα) or genotoxic stressed conditions, this process involves the linear polyubiquitination of NEMO catalyzed by the linear ubiquitin chain assembly complex (LUBAC) (11–14). LUBAC is the only E3 ligase complex capable of synthesizing methionine 1 (Met1/M1)–linked ubiquitin chains in a head-to-tail manner in mammalian cells (15). The complex is composed of three subunits: heme-oxidized IRP2 ubiquitin ligase 1L (HOIL-1L), HOIL-interacting protein (HOIP), and SHANK-associated RH domain interacting protein (SHARPIN), each of which exhibits specific functions. The HOIP subunit contains all the catalytic machinery to synthesize M1-linked chains of ubiquitin.

Among the 100 distinct deubiquitinases (DUBs) identified, three are reportedly involved in the negative regulation of NF-κB signaling: ovarian tumor family deubiquitinase OTULIN (also known as FAM105B), cylindromatosis (CYLD), and tumor suppressor A20 (16–18). OTULIN is the only identified DUB displaying the highest specificity, which exclusively cleaves linear (Met1-linked) polyubiquitin chains (19–21). OTULIN has been reported to interact with the LUBAC complex via HOIP to counteract NF-κB activation upon TNFα stimulation (22, 23). OTULIN has multiple functions in controlling embryonic development, cell death, inflammation, and innate immune signaling (24–27). Its biallelic hypomorphic mutations led to an early-onset autoinflammatory disease named otulipenia (28). Of note, severe inflammatory and autoimmune or immunodeficient diseases result from gene mutations or haploinsufficiency of LUBAC subunits or the identified DUBs (29–32). This process underscores the importance of the ubiquitin assembling–disassembling mechanisms in autoimmunity, inflammation, and infection.

Here, we employed cellular models and clinical specimens of triple-negative breast cancer (TNBC), the most lethal subtype of breast cancer owing to high heterogeneity and aggressive nature. Chemoresistance greatly hinders the effective use of chemotherapy drugs in TNBC, which is mainly attributed to the genotoxic NF-κB activation (33). We discovered molecular events on OTULIN underpinning the genotoxic NF-κB activation, which can be generalized to TNFα-induced inflammatory response.

## Results

### OTULIN Counteracts LUBAC in Regulating Genotoxic NF-κB Activation.

**Fig. 1.**
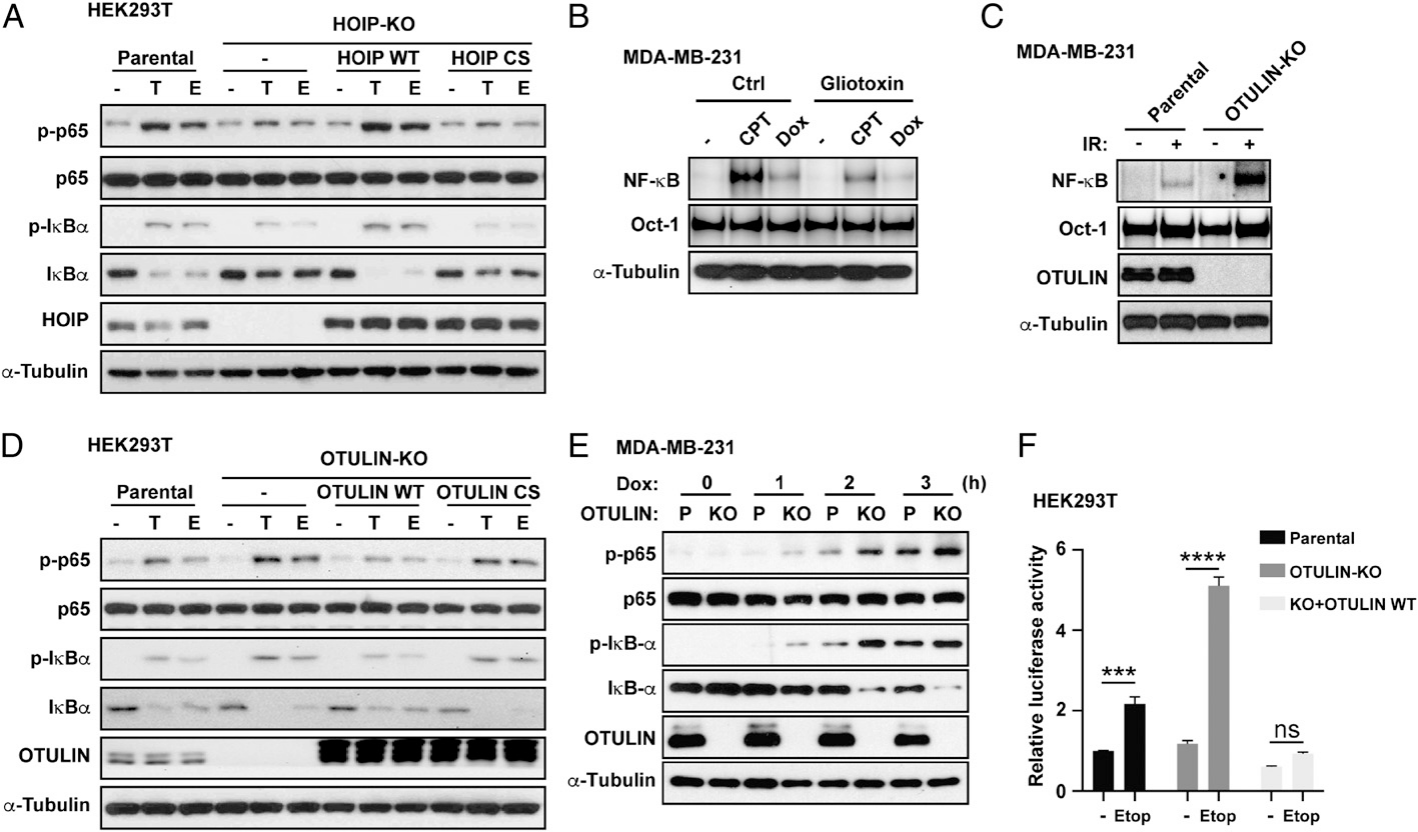
LUBAC (HOIP) and OTULIN play opposing roles in genotoxic NF-κB activation. (*A*) HOIP-KO HEK293T cells were reconstituted with an HOIP WT or C885S mutant. After transfection for 48 h, cells were treated with TNFα (T, 10 ng/mL, 15 min) or Etop (E, 10 μM, 2 h). Whole-cell lysates were collected later and subjected to immunoblotting with antibodies as indicated. (*B*) Gel shift analysis of NF-κB activation by using Igκ probe in MDA-MB-231 cells pretreated with or without LUBAC inhibitor gliotoxin (1 μM) for 24 h and subsequently treated with CPT (10 μM, 2 h) or Dox (2 μg/mL, 2 h). Total cell lysates were analyzed by immunoblotting with antibody against α-tubulin. (*C*) Parental and OTULIN-KO MDA-MB-231 cells were exposed to IR (10 Gy, +) or were not (-), then were left in the incubator for 2 h. NF-κB activation and protein expression levels of cells were analyzed by gel shift assay and immunoblotting with antibodies as indicated. (*D*) OTULIN-KO HEK293T cells were reconstituted with OTULIN WT or C129S mutant. After transfection for 48 h, cells were treated with TNFα (T, 10 ng/mL, 15 min) or Etop (E, 10 μM, 2 h). Whole-cell lysates were collected later and subjected to immunoblotting with antibodies as indicated. (*E*) Immunoblotting analysis of protein expression levels in parental (P) and OTULIN-KO MDA-MB-231 cells treated with Dox (2 μg/mL) for indicated times. (*F*) Parental, OTULIN-KO, and OTULIN-reconstituted HEK293T cells were cotransfected with κB-Fluc/hRluc-TK reporter constructs. After transfection for 24 h, cells were treated with or without Etop (10 μM, 6 h), and cell extracts were prepared to determine luciferase activity. The activities of firefly and Renilla luciferases were evaluated sequentially from a single sample in the dual-luciferase reporter assay. Results were plotted as firefly normalized to Renilla luciferase activity. The relative luciferase activities compared with the control (lane 1) are shown as mean ± SEM from the experiments performed in triplicate. Two-way ANOVA followed by Tukey’s posthoc test was used to determine statistical significance for multiple comparisons. ***P < 0.001; ****P < 0.0001; ns, not significant.

It is well demonstrated that NF-κB signaling is activated under genotoxic stressed conditions (10, 14). LUBAC-dependent linear ubiquitination of NEMO has been demonstrated as a critical process of atypical NF-κB activation upon genotoxic stress in which linear ubiquitin chains serve as signaling scaffolds to promote IKKβ phosphorylation by transforming growth factor β-activated kinase 1 (TAK1) (14, 34–36). Here we provide additional evidence supporting a LUBAC-dependent mechanism underlying the genotoxic NF-κB signaling activation by investigating the LUBAC subunits of HOIP, HOIL-1L, and SHARPIN. First, we found that the chemotherapy drug etoposide (Etop)- and TNFα-induced NF-κB activation, as indicated by the phosphorylation of p65 (p-p65) and IκBα (p-IκBα), and the subsequent degradation of IκBα, was substantially attenuated in three independent clones of HOIP-knockout (KO) 293T cells generated by the CRISPR/Cas9 system (37); representative data of one clone are shown in Fig. 1*A*. Reconstitution of the HOIP wild-type (WT) or the catalytically inactive mutant HOIP C885S (38) in HOIP-KO cells revealed the reliance of NF-κB activation on the HOIP catalytic activity (Fig. 1*A*). Gel shift assay indicated that genotoxic and inflammatory NF-κB activation was attenuated in HOIP-KO 293T clones, HOIL-1^−/−^ mouse embryonic fibroblasts (MEFs), and SHARPIN-null chronic proliferative dermatitis (cpdm) MEFs (*SI Appendix*, Fig. S1 *A–C*). NF-κB activation was increased gradually upon camptothecin (CPT) treatment and peaked at 2 h, which was reduced in HOIL-1^−/−^ MEF cells at all the indicated times (*SI Appendix*, Fig. S1*D*). Furthermore, we found that a highly bioactive fungal metabolite gliotoxin with its well-known immunosuppressive action as a functional NF-κB inhibitor attenuated genotoxic NF-κB activation by inhibiting LUBAC (Fig. 1*B*), consistent with its new identity as a selective binder to the really interesting new gene (RING)-between-RING (RBR) domain of the HOIP, the catalytic core unit of LUBAC (39). Collectively, all the subunits of LUBAC play essential roles in genotoxic NF-κB activation.

OTULIN exclusively cleaves linear polyubiquitin chains that are conjugated by LUBAC. To test whether OTULIN regulates LUBAC-mediated genotoxic NF-κB activation, we also generated three clones of OTULIN-KO in the HEK293T and MDA-MB-231 cells (namely OTULIN-KO HEK293T and OTULIN-KO MDA-MB-231). We found a substantially up-regulated signal of linear polyubiquitination in both OTULIN-KO HEK293T and MDA-MB-231 clones without LUBAC degradation (*SI Appendix*, Fig. S2 *A* and *B*). Furthermore, NF-κB activation was dramatically augmented by OTULIN KO as measured by gel shift assay in OTULIN-KO MDA-MB-231 or HEK293T cells compared with the parental cells upon various stimuli, including genotoxic ionizing radiation (IR), doxorubicin (Dox), carboplatin (CBP), CPT, and proinflammatory TNFα (Fig. 1*C* and *SI Appendix*, Fig. S1 *E* and *F*). Furthermore, the augmented NF-κB activation in the OTULIN-KO cells was fully prevented by the reconstitution of OTULIN WT but not by the reconstitution with its catalytically inactive C129S mutant (Fig. 1*D*). In a time-course study, Dox induced markedly increased p-p65 and p-IκB-α after the 2 h treatment in OTULIN-KO cells (Fig. 1*E*). In addition, treatment with Etop also induced much more robust NF-κB activation based on a dual-luciferase NF-κB–dependent reporter assay in OTULIN-KO cells compared with parental cells, which was attenuated by the reconstitution of OTULIN WT (Fig. 1*F*).

To investigate how OTULIN negatively regulates genotoxic NF-κB activation, we next examined the NF-κB modulator NEMO, which is known to be linearly ubiquitinated by LUBAC under genotoxic stress (14). As expected, NEMO linear ubiquitination was induced by the treatments of Etop and Dox, which were suppressed by OTULIN overexpression (*SI Appendix*, Fig. S2*C*). On the contrary, in OTULIN-KO cells, linear ubiquitination levels of NEMO were further augmented (*SI Appendix*, Fig. S2*D*). Taken together, these results indicate that genotoxic NF-κB activation is counterbalanced by LUBAC and OTULIN, each part plays a crucial role. In the subsequent work, we intended to investigate the interplay between LUBAC-mediated linear ubiquitination versus OTULIN-mediated deubiquitination events under both normal unstressed and genotoxic stressed conditions.

### The N-terminal Domain of OTULIN Interacts with HOIP-PUB Facilitated by Linear Ubiquitination.

The N-terminal PNGase/ubiquitin-associated (UBA) or UBX (PUB) domain of the LUBAC component HOIP is highly conserved in several proteins, including PNGase (40). PNGase-PUB electrostatically binds to ubiquitin chains and the ubiquitin-like (UBL) domain of HR23 (41). In addition, the ubiquitin-associated domain of HOIP specifically interacts with the UBL domain of HOIL-1L, which is crucial for the LUBAC formation (42). HOIP also comprises the Npl4 zinc finger (NZF) ubiquitin-binding domains that guide LUBAC to ubiquitinated proteins (23). We therefore speculated that HOIP might interact with linear ubiquitin chains of OTULIN via some of these ubiquitin-binding domains, which would enhance the affinity between OTULIN and HOIP. It has been reported that the PUB-interacting motif (PIM) of OTULIN interacts with the N-terminal PUB domain of the LUBAC component HOIP (22, 23). To validate the importance of these interactions in cancer models, we first determined the specific domains of OTULIN and HOIP interaction under normal unstressed conditions by using a domain truncation strategy (*SI Appendix*, Fig. S3*A*).

To test the hypothesis, truncated HOIP moieties expressed with a Myc tag were cotransfected with hemagglutinin (HA)-OTULIN into 293T cells. Co-immunoprecipitation (Co-IP) showed that the PUB-deleted variants exclusively abolished the interaction between OTULIN and HOIP, indicating that only the PUB domain was indeed involved in OTULIN interaction (*SI Appendix*, Fig. S3*B*). By in vitro pulldown assay using recombinant proteins expressed with His and GST tags, we further demonstrated that when fused with a head-to-tail linked tetra-ubiquitin (Ub4) construct, the N-terminal domain of OTULIN (i.e., 1–80aa) further strengthened its binding to the His-PUB (*SI Appendix*, Fig. S3*C*), suggesting that OTULIN’s linear ubiquitin chains are the preferred binding partner to the HOIP-PUB. As shown in the aligned sequences of the PNGase- and HOIP-PUB domains (*SI Appendix*, Fig. S3*D*), we also identified the two corresponding residues Asp117 (D) and Glu127 (E), which were previously shown to be critical for the PNGase-PUB interaction with HR23-UBL and ubiquitin chains (41), required for the interaction between HOIP-PUB and Ub4/*N*-OTULIN-Ub4 (*SI Appendix*, Fig. S3 *E* and *F*). The abolishing effects of the double-mutation variant (D117A and E127A) were further confirmed in the 293T cell system (*SI Appendix*, Fig. S3*G*). We concluded that the linear ubiquitin chains of OTULIN promoted its binding to HOIP through interacting with the PUB domain.

### OTULIN Is Linearly Ubiquitinated by LUBAC/HOIP on K64/66 under Unstressed Conditions.

**Fig. 2.**
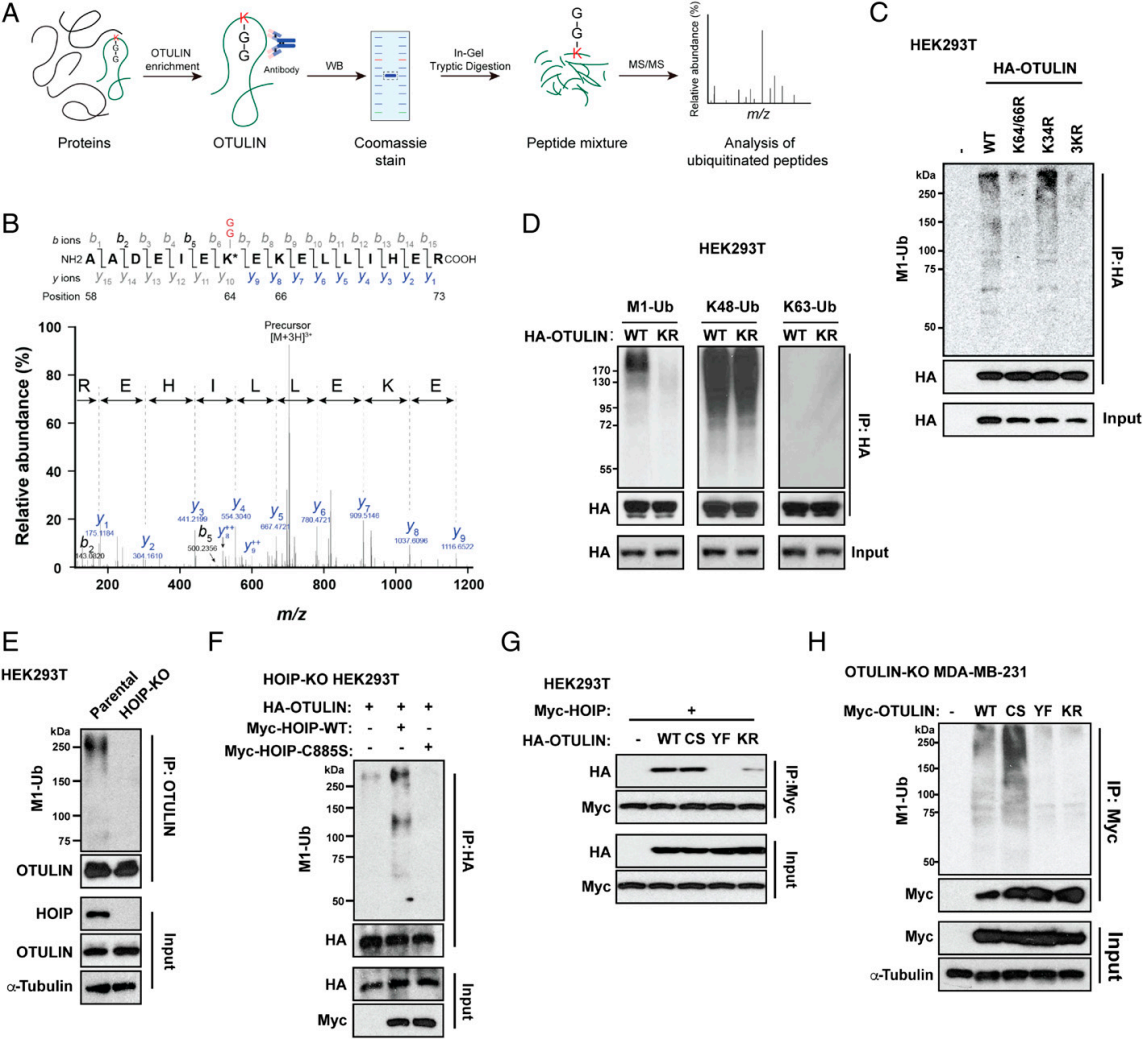
OTULIN is linearly ubiquitinated by LUBAC (HOIP), which is required for the OTULIN–LUBAC/HOIP interaction under an unstressed condition. (*A*) Workflow of the identification of ubiquitination (GG)-modified peptides of enriched OTULIN. (*B*) MS/MS spectrum showing the identification of an OTULIN peptide carrying ubiquitination (GG) on lysine (K) 64. *Top*, MS/MS fragmentation of the peptide (AADEIEK(-GG)EKELLIHER). *Bottom*, a series of black (b) and blue (y) product ions are detected in the 3-charge MS/MS spectrum, providing high confidence in the identification and localization of the ubiquitin modification. The *x* axis represents the mass-to-charge (*m/z*) ratio, and the *y* axis shows the relative intensity of different peaks. The monoisotopic precursor mass value [M+3H]^3+^ of the ubiquitinated peptide is shown. (*C*) Linear ubiquitination of immunoprecipitated HA-OTULIN WT, K64/66R, K34R, and K34/64/66R mutants in HEK293T cells. HEK293T cells were transfected with HA-OTULIN WT or indicated mutants. After 48 h, cell lysates were immunoprecipitated with anti-HA antibody and immunoblotted with anti-linear ubiquitin and anti-HA antibodies. (*D*) Different types of ubiquitination of immunoprecipitated HA-OTULIN WT and K64/66R mutant in HEK293T cells. HEK293T cells were transfected with HA-OTULIN WT or its K64/66R mutant. After 48 h, cell lysates were immunoprecipitated with anti-HA antibody and immunoblotted with antibodies for M1-Ub chains, K48-Ub chains, K63-Ub chains, and HA-tag (hemagglutinin tag). (*E*) Linear ubiquitination of immunoprecipitated endogenous OTULIN in parental and HOIP-KO HEK293T cells. Cell lysates were immunoprecipitated with anti-OTULIN antibody and immunoblotted with indicated antibodies. (*F*) Linear ubiquitination of immunoprecipitated HA-OTULIN in HEK293T cells. HOIP-KO HEK293T cells were cotransfected with HA-OTULIN and Myc-HOIP WT or its C885S mutant. After 48 h, cell lysates were immunoprecipitated with anti-HA antibody and immunoblotted with indicated antibodies. (*G*) Co-IP analysis of the interaction between Myc-HOIP and HA-OTULIN WT, C129S, Y56F, or K64/66R mutant in HEK293T cells. HEK293T cells were cotransfected with Myc-HOIP and indicated HA-OTULIN constructs in HEK293T cells. After 48 h, cell lysates were immunoprecipitated with anti-Myc antibody and immunoblotted with antibodies for HA-tag and Myc-tag. (*H*) Linear ubiquitination of immunoprecipitated Myc-OTULIN in OTULIN-KO MDA-MB-231 cells. Cells were transfected with or without Myc-OTULIN WT, C129S, Y56F, or K64/66R mutants. After 48 h, cell lysates were immunoprecipitated with anti-Myc antibody and immunoblotted with the indicated antibodies. (+) indicates the samples receiving the specific transfection listed on the left side, and (-) indicates mock controls.

To date, LUBAC is the only known E3 ligase that specifically generates linear ubiquitin chains. The interaction between LUBAC and OTULIN led us to speculate that OTULIN might be linearly ubiquitinated by LUBAC. To confirm this hypothesis, we performed a mass spectrometry (MS)–based proteomics experiment on the enriched OTULIN protein (Fig. 2*A*). The resulting MS data were searched against the protein database using SEQUEST. We identified a total of eight ubiquitinated OTULIN residues, including K64 and K66. An MS/MS spectrum supports high confidence in the identification and localization of the OTULIN ubiquitin modification on K64 (Fig. 2*B*). We also detected ubiquitin modification on K66, albeit to a lesser degree; none was detected on K34 (*SI Appendix*, *Materials and Methods*). Of note, the MS study could only reveal the ubiquitination sites of OTULIN but could not distinguish between linear or other Lys-dependent ubiquitin types. Hence, we also performed an IP assay to determine the ubiquitin types of OTULIN on K64/66. M1-linked linear ubiquitination on OTULIN was only detected in cells expressing the WT or K34R mutant, but not in the OTULIN K64/66R mutant (Fig. 2*C*). We also investigated whether K64/66 is responsible for other types of ubiquitination. K48 ubiquitination of OTULIN was not affected by the mutation of K64/66R. Since we did not observe any K63 signal of OTULIN, these results indicate that K64/66 residues are critical for OTULIN linear ubiquitination (Fig. 2*D*).

Next, we tested whether the linear ubiquitination of OTULIN is LUBAC-dependent. It is known that only the RBR domain of HOIP, but not HOIL-1L, is necessary for LUBAC to form linear ubiquitin chains, despite the fact that both HOIP and HOIL-1L are considered the E3 ligases of the RBR family (38, 42). Consequently, only the loss of HOIP substantially abolishes LUBAC’s ligase activity. Therefore, we determined the linear ubiquitination of OTULIN in parental and HOIP-KO 293T cells. Indeed, OTULIN could be linearly ubiquitinated in the parental cells, and the M1–ubiquitin signal was almost nondetectable in the absence of HOIP (Fig. 2*E*). To mitigate off-target effects, we reconstituted HOIP WT and inactive HOIP C885S mutant in HOIP-KO 293T cells. OTULIN linear ubiquitination was only rescued by HOIP WT (Fig. 2*F*), suggesting that HOIP’s RBR E3 ligase activity is required for OTULIN linear ubiquitination.

### Linear Ubiquitination of OTULIN Is Required for the Interaction with LUBAC and the Genotoxic NF-κB Inhibition.

We next determined the impacts of several OTULIN mutants in their abilities to interact with LUBAC and to counteract genotoxic NF-κB activation. C129S (CS) and Y56F (YF) represent two known loss-of-function mutants: The former represents a catalytically inactive OTULIN (19) while Tyr56 phosphorylation is known to lead to the disruption of the OTULIN–LUBAC interaction, which can be manifested by the YF mutation (22, 23). Of note, three independent Co-IP assays showed a complete disruption of HOIP binding to the OTULIN Y56F mutant, consistent with previous studies, while the OTULIN K64/66R mutant lost its interaction with LUBAC by 81%, compared with the interaction with OTULIN WT (Fig. 2*G*), indicating that the physical interaction between OTULIN and LUBAC requires and is stabilized by the OTULIN linear ubiquitin chains. Interestingly, CS mutation of OTULIN did not affect its interaction with LUBAC (Fig. 2*G*) nor the levels of OTULIN linear ubiquitination (Fig. 2*H*). All three mutants lost their counteracting roles in genotoxic and inflammatory NF-κB activation (*SI Appendix*, Fig. S4 *A–F*). Consistently, reconstitution of the OTULIN K64/66R mutant did not inhibit NF-κB activation upon ETOP and TNFα treatment (*SI Appendix*, Fig. S4*I*). To verify the loss function of the OTULIN K64/66R mutation in regulating the NF-κB pathway, we compared the kinetics between OTULIN-WT– and KR-reconstituted groups. OTULIN K64/66R cells displayed higher NF-κB activity without changing its kinetics (i.e., the rates of its activation and resolution; *SI Appendix*, Fig. S4 *G* and *H*). Although the mutation at K64/66 did not alter the enzymatic activity of OTULIN, it failed to inhibit NF-κB activation due to a lack of LUBAC interaction. On the other hand, direct determination of cell death also confirmed that OTULIN-KO MDA-MB-231 cells reconstituted with either the Y56F or K64/66R mutant OTULIN both resulted in increased cancer cell survival under genotoxic stress via dysregulated NF-κB signaling since these effects were abolished by overexpressing an IκBα superrepressor (IκBα SR) (43) or cotreatment with an IKKβ inhibitor TPCA-1 (*SI Appendix*, Fig. S5). These findings indicate that the linear ubiquitin chains of OTULIN are required for OTULIN’s counteracting role in genotoxic NF-κB activation, which depends on the physical interaction between OTULIN and LUBAC.

### Genotoxic Stress Induces OTULIN Dimerization and Self-Deubiquitination Required for NF-κB Activation.

**Fig. 3.**
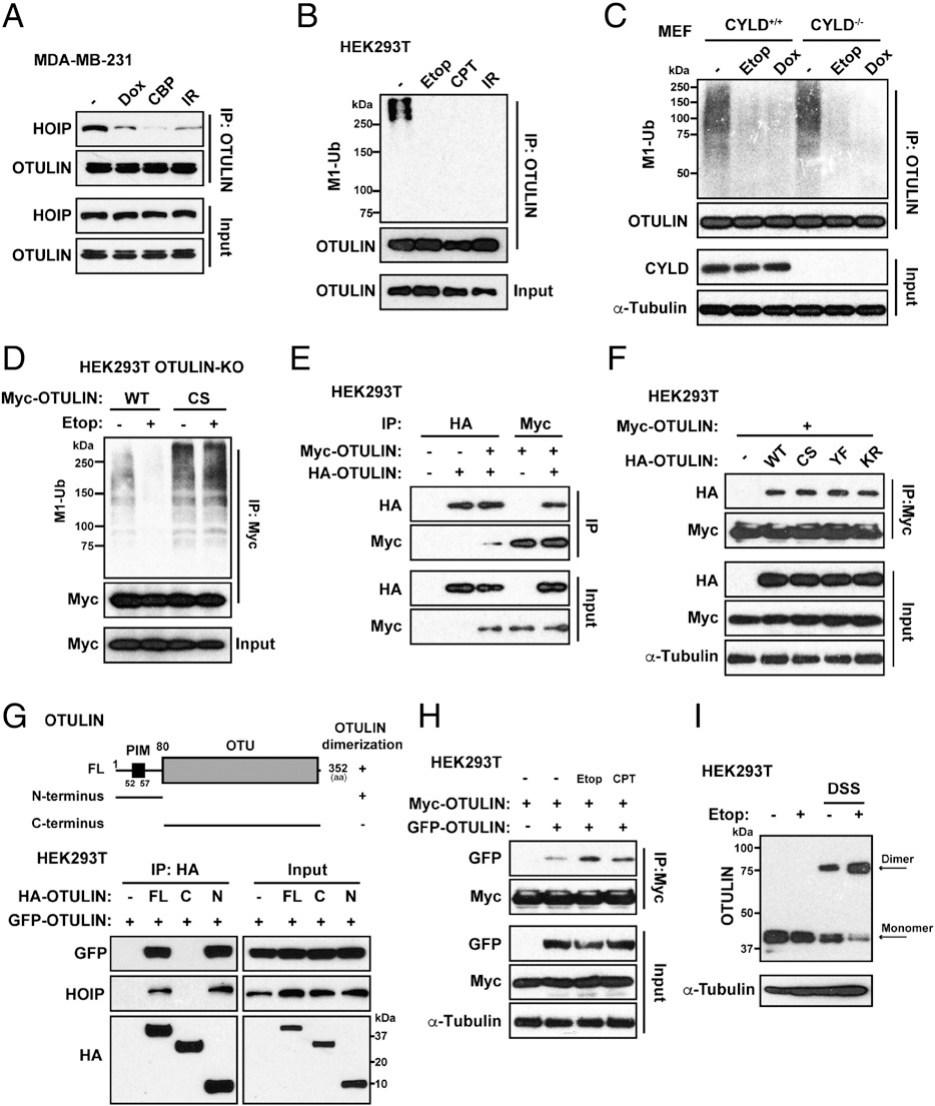
Genotoxic stress induces OTULIN dimerization, OTULIN self-deubiquitination, and the OTULIN–HOIP complex dissociation. (*A*) Co-IP analysis of the interaction between endogenous OTULIN and HOIP in MDA-MB-231 cells treated with dimethyl sulfoxide (DMSO) (-), Dox (2 μg/mL, 2h), CBP (10 μg/mL, 2h) or exposed to IR (10 Gy) and left in the incubator for 2 h. Cell lysates were immunoprecipitated with anti-OTULIN antibody and immunoblotted with antibodies for HOIP and OTULIN. (*B*) Linear ubiquitination of immunoprecipitated endogenous OTULIN in HEK293T cells treated with DMSO (-), Etop (10 μM, 2h), CPT (10 μM, 2h) or exposed to IR (10 Gy) and left in the incubator for 2 h. Cell lysates were immunoprecipitated with anti-OTULIN antibody and immunoblotted with anti-linear ubiquitin and anti-OTULIN antibodies. (*C*) Linear ubiquitination of immunoprecipitated endogenous OTULIN in CYLD^+/+^ and CYLD^−/−^ MEFs treated with Etop (10 μM) or Dox (2 μg/mL) for 2 h or left untreated (-). Cell lysates were immunoprecipitated with anti-OTULIN antibody and immunoblotted with indicated antibodies. (*D*) Linear ubiquitination of immunoprecipitated Myc-OTULIN in HEK293T cells. OTULIN-KO HEK293T cells were reconstituted with Myc-OTULIN WT or its C129S mutant. After 48 h, cells were treated with or without Etop (10 μM, 2 h). Cell lysates were immunoprecipitated with anti-Myc antibody and immunoblotted with anti-linear ubiquitin and anti-Myc antibodies. (*E*) Co-IP analysis of the interaction between Myc-OTULIN and HA-OTULIN in HEK293T cells. Cells were cotransfected with Myc-OTULIN and HA-OTULIN as indicated. After 48 h, cell lysates were immunoprecipitated with anti-HA or anti-Myc antibody and immunoblotted with indicated antibodies. (*F*) Co-IP analysis of the interaction between Myc-OTULIN and HA-OTULIN in HEK293T cells. Cells were cotransfected with Myc-OTULIN and HA-OTULIN WT, C129S, Y56F, or the K64/66R mutant. After 48 h, cell lysates were immunoprecipitated with anti-Myc antibody and immunoblotted with antibodies as indicated. (*G*) *Top*, mapping of OTULIN domain required for the OTULIN dimerization. *Bottom*, interactions between GFP-OTULIN and indicated HA-OTULIN constructs were analyzed by Co-IP assay in HEK293T cells. Cells were cotransfected with GFP-OTULIN and full-length (FL) HA-OTULIN, C terminus (C) of OTULIN, or N terminus (N) of OTULIN. After 48 h, cell lysates were immunoprecipitated with anti-HA antibody and immunoblotted with indicated antibodies. (*H*) Co-IP analysis of the interaction between Myc-OTULIN and GFP-OTULIN in HEK293T cells. Cells were cotransfected with Myc-OTULIN and GFP-OTULIN as indicated for 48 h and subsequently treated with Etop (10 μM, 2 h) or CPT (10 μM) or left untreated (-). Cell lysates were immunoprecipitated with anti-Myc antibody and immunoblotted with indicated antibodies. (*I*) DSS cross-linking assay reveals the formation of OTULIN dimers by Western blot analysis. HEK293T cells were treated with or without Etop (10 μM, 2 h) followed by the cross-linking assay. Cells were collected and incubated in 500 μL cold PBS containing DSS (1 mM) for 30 min at RT, which was quenched by 10 mM Tris⋅HCl (pH 7.5) for 15 min at RT. Cells were obtained by 200 g centrifugation for cell lysis. Cell lysates were immunoblotted with indicated antibodies. (+) indicates the samples receiving the specific treatment/transfection listed on the left side, and (-) indicates mock controls.

These results led us to reason that physical interaction between OTULIN and LUBAC must be disrupted by genotoxic stress. Indeed, the various chemotherapeutic agents tested all induced nearly complete loss of the physical interaction between OTULIN and HOIP (Fig. 3*A*), which correlated with a complete loss of linear ubiquitinated OTULIN (Fig. 3*B*). Thus far, OTULIN and CYLD are the only known DUBs that reportedly cleave linear ubiquitin chains in vivo (16). To test whether CYLD plays a role in OTULIN deubiquitination, we determined the linear ubiquitinated state of OTULIN in CYLD-null cells (CYLD^−/−^) and found no effect compared with that in the CYLD^+/+^ cells (Fig. 3*C*). Interestingly, the OTULIN C129S mutant displayed higher levels of Met1–ubiquitin modification (Fig. 3*D*). Therefore, we reasoned that the genotoxic stress–induced deubiquitination of OTULIN may be caused by OTULIN itself.

To prove whether OTULIN is responsible for its deubiquitination upon genotoxic stress, we undertook several approaches. We first investigated whether OTULIN proteins can interact among themselves and undergo self-deubiquitination of each other. Co-IP assay showed that OTULIN expressed with two different tags such as as Myc and HA could interact in 293T cells (Fig. 3*E*); this interaction was not affected by any of the mutants such as CS, YF, or KR (Fig. 3*F*). Of note, it is the PIM-containing N-terminal domains of OTULIN (i.e., 1-80aa), but not the C-terminal domains, that can interact with each other (Fig. 3*G*). Although OTULIN proteins overexpressed with two different tags in 293T cells could be coimmunoprecipitated, their interactions were further enhanced under genotoxic stressed conditions such as Etop and CPT (Fig. 3*H*). Consistently, in the presence of the DSS cross-linker, we detected an increased formation of OTULIN dimers upon genotoxic Etop treatment (Fig. 3*I*).

**Fig. 4.**
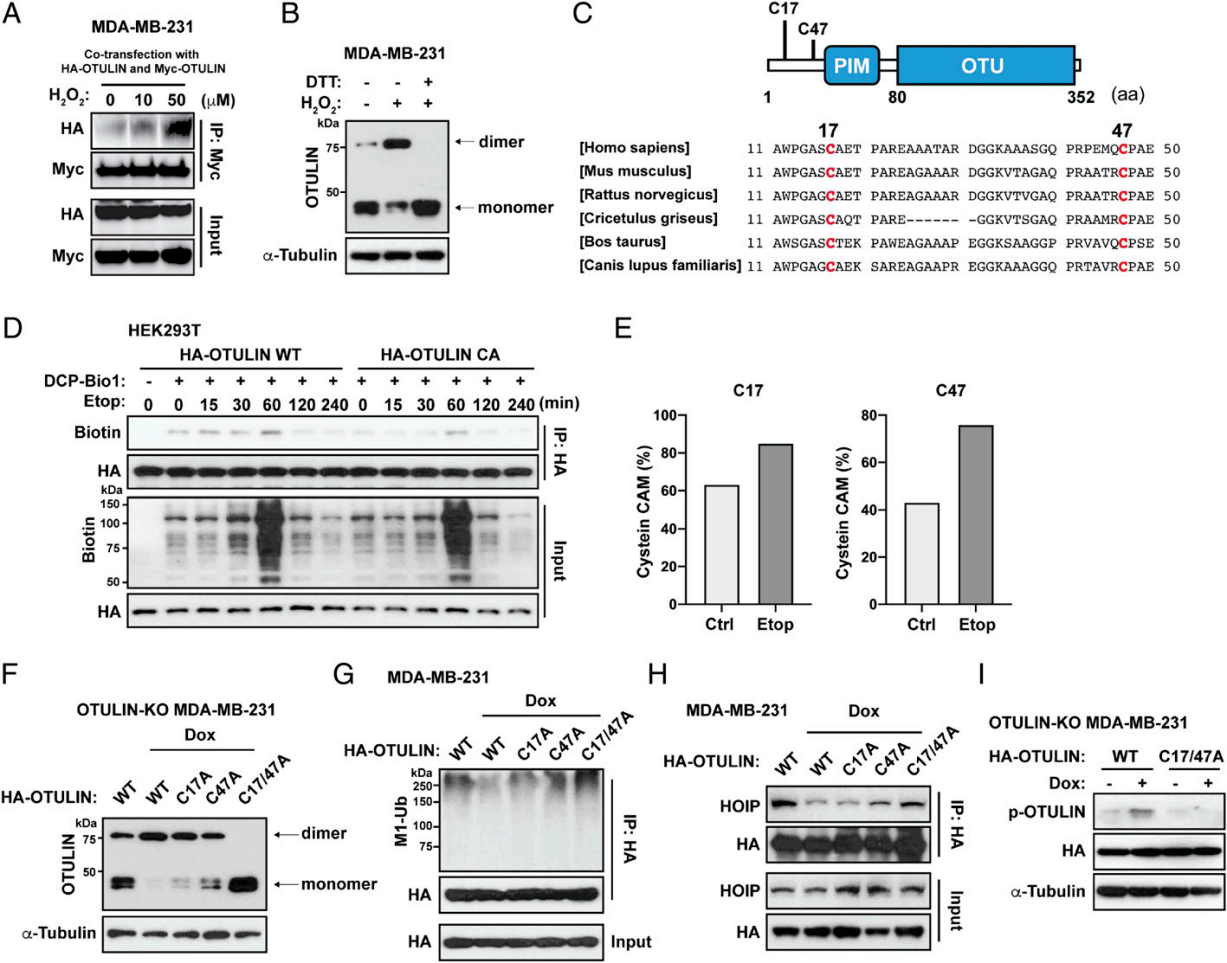
Oxidative stress mediates OTULIN dimerization by forming disulfide bonds via C17 and C47. (*A*) Co-IP analysis of the interaction between Myc-OTULIN and HA-OTULIN in MDA-MB-231. After the cotransfection with Myc-OTULIN and HA-OTULIN for 48 h, cells were treated with H_2_O_2_ (0, 10, or 50 μM) for 15 min. Cell lysates were immunoprecipitated with anti-Myc antibody and immunoblotted with anti-HA and anti-Myc antibodies. (*B*) Western blot analysis of OTULIN dimerization in MDA-MB-231 cells. Cells were treated with (+) or without (-) H_2_O_2_ (50 μM, 15 min), subjected to cell lysis with or without DTT (10 mM), and boiled with SDS loading buffer (no 2-ME). Cell lysates were immunoblotted with indicated antibodies. (*C*) *Top*, the locations of C17 and C47 in OTULIN. *Bottom*, the amino acid sequence alignments of the OTULIN N terminus (i.e., 11–50aa) shows the conservation of C17 and C47 in various species. (*D*) Immunoprecipitation analysis of the biotin-labeled HA-OTULIN (WT or C17/47A) in HEK293T cells treated with Etop (2 μM) for indicated time points and lysed with DCP-Bio1 (+) or wthout (-) as described in *Materials and Methods*. Cell lysates were immunoprecipitated with anti-HA antibody and immunoblotted with indicated antibodies. (*E*) Liquid chromatography-tandem MS analysis of Cys alkylation. HEK293T was treated with Etop (2 μM) for 2 h. The percentage of IAM-labeled Cys was calculated by the abundances of Cys with carbamidomethyl divided by the total abundances of corresponding Cys (× 100%). (*F*) Western blot analysis of OTULIN dimerization. OTULIN-KO MDA-MB-231 cells were reconstituted with indicated OTULIN constructs (48 h) and then treated with Dox (2 μg/mL, 2 h). Cell lysates were immunoblotted by indicated antibodies without DTT or 2-ME. (*G*) Linear ubiquitination of immunoprecipitated HA-OTULIN in MDA-MB-231 cells treated as in *F*. Cell lysates were immunoprecipitated with anti-HA antibody and immunoblotted with indicated antibodies. (*H*) Co-IP analysis of the interaction between HOIP and HA-OTULIN in MDA-MB-231 cells treated as in *F*. Cell lysates were immunoprecipitated with anti-HA antibody and immunoblotted with anti-HA and anti-HOIP antibodies. (*I*) Western blot analysis of OTULIN Tyr56 phosphorylation. OTULIN-KO MDA-MB-231 cells were reconstituted with HA-OTULIN WT or its C17/47A mutant (48 h) and then treated with (+) or without (-) Dox (2 μg/mL, 2 h). Cell lysates were immunoblotted with indicated antibodies.

**Fig. 5.**
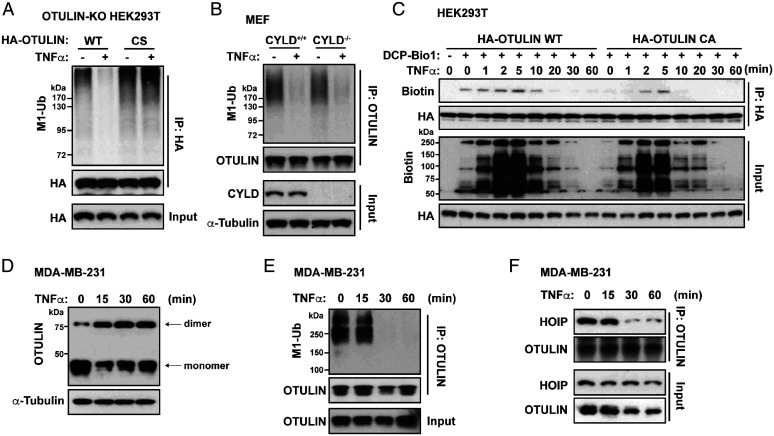
Analysis of OTULIN dimers, linear ubiquitination, and OTULIN-HOIP interaction in TNFα-treated cells. (*A*) Linear ubiquitination of immunoprecipitated HA-OTULIN. OTULIN-KO HEK293T cells were reconstituted with HA-OTULIN WT or C129S mutant (48 h) and then treated with (+) or without (-) TNFα (10 ng/mL, 15 min). Cell lysates were immunoprecipitated with anti-HA antibody and immunoblotted with indicated antibodies. (*B*) Linear ubiquitination of immunoprecipitated OTULIN in CYLD^+/+^ and CYLD^−/−^ cells treated as in *A*. Cell lysates were immunoprecipitated with anti-OTULIN antibody and immunoblotted with indicated antibodies. (*C*) Immunoprecipitation analysis of the biotin-labeled HA-OTULIN (WT or C17/47A) in HEK293T cells treated with TNFα (10 ng/mL) for indicated time points and lysed with DCP-Bio1 (+) or without (-) as described in *Materials and Methods*. Cell lysates were immunoprecipitated with anti-HA antibody and immunoblotted with indicated antibodies. (*D*) Western blot analysis of OTULIN dimerization. MDA-MB-231 cells were treated with TNFα (10 ng/mL) for indicated time points. Cell lysates were immunoblotted by indicated antibodies without DTT or 2-ME. (*E*) Linear ubiquitination of immunoprecipitated OTULIN in MDA-MB-231 cells treated as in *D*. Cell lysates were immunoprecipitated with anti-OTULIN antibody and immunoblotted with indicated antibodies. (*F*) Co-IP analysis of the interaction between OTULIN and HOIP in MDA-MB-231 cells treated as in *D*. Cell lysates were immunoprecipitated with anti-OTULIN antibody and immunoblotted with antibodies for OTULIN and HOIP.

Oxidative stress is a common mediator of apoptotic and genotoxic signaling, which often involves posttranslational modifications on critical cysteine (Cys) residues to alter the conformational structure and functions of a protein. Indeed, we found that treatment of MDA-MB-231 cells by hydrogen peroxide (H_2_O_2_) can facilitate intermolecular OTULIN interaction and induce the formation of OTULIN dimers in a dose-dependent manner (Fig. 4*A*); the nature of the dithiothreitol (DTT)-sensitive dimers (Fig. 4*B*) suggests the involvement of covalent disulfide bonds. We next investigated the impacts of mutagenized Cys residues in the N-terminal domain of OTULIN (Cys17 and Cys47), which are conserved among different species (Fig. 4*C*). Using 3-(2,4-dioxocyclohexyl) propyl appended to biotin (DCP-Bio1) to label Cys sulfenic acid of enriched OTULIN, we observed that the initial stage of the OTULIN oxidation was rapidly induced by Etop and TNFα, temporally peaking at 1 h and 5 min, respectively. This was followed by the restoration of the cysteine sulfenic acid production to baseline levels, which indicates the continued progression of oxidation (i.e., overoxidation) based on the universal kinetic curve detected/reported using this DCP-Bio1 (44). Of note, we detected lower levels of initial oxidation in the OTULIN C17/47A mutant at all the time points compared with that of OTULIN WT, indicating that the Cys17/47 residues of OTULIN largely mediate the OTULIN oxidation (Figs. 4*D* and 5*C*). To further verify the Cys17/47 oxidation via MS, we also labeled reduced Cys by *N*-ethylmaleimide and labeled oxidized Cys (e.g., disulfide, S-glutathionylation) by iodoacetamide (IAM) after DTT reduction (*SI Appendix*, Fig. S4*J*). The ratios of the C17 and the C47 that reacted with IAM (i.e., carbamidomethylation) were significantly increased upon Etop treatment in HEK293T cells (Fig. 4*E*), indicating that Cys17/47 of OTULIN can be oxidized and increasingly oxidized during VP16 treatment. Either of the OTULIN Cys mutants resulted in a more than 40% reduction (C17A, 44.4%; C47A, 66.3%; *n* = 3 independent experiments) of the dimer formation upon Dox stress compared with WT; the double-Cys mutant OTULIN completely abolished dimer formation upon Dox (Fig. 4*F*). Moreover, the degrees of dimer formation negatively correlated with the linear ubiquitinated OTULIN (Fig. 4*G*) and the physical interaction between OTULIN and HOIP (Fig. 4*H*). Interestingly, Cys17/47 mutant OTULIN proteins lost the capability to be phosphorylated on the Tyr56 site upon Dox treatment (Fig. 4*I*). Altogether, these findings reveal an important role of OTULIN dimerization as one underlying mechanism of the loss of function of OTULIN during the genotoxic response.

### OTULIN Plays Similar Roles under Inflammatory Conditions (TNFα).

OTULIN’s negative regulation on NF-κB signaling has been most studied in the experimental settings of proinflammatory stimuli such as TNFα (22–26). To determine whether the several molecular events on OTULIN we identified under normal and genotoxic stressed conditions can be generalized to the receptor-mediated immune complex response, we repeated key experimental assays in HEK293T and MDA-MB-231 cells treated with TNFα in a time-course study. We observed similar results, showing that OTULIN oxidation at Cys17/47 and OTULIN dimer formation negatively correlated with OTULIN linear ubiquitination and OTULIN-HOIP interaction during TNFα treatment (Fig. 5 *C*–*F*). In addition, TNFα-induced OTULIN deubiquitination was also dependent on OTULIN (Fig. 5 *A* and *B*). To test the functional effect of OTULIN Cys17/47, we reconstructed the OTULIN C17/47A mutant in OTULIN-KO 293T cells and observed a stronger inhibitory effect on NF-κB activation upon TNFα or Etop treatment (*SI Appendix*, Fig. S4*I*). The data are consistent with our model: that the OTULIN C17/47A mutant, which is incapable of dimerization, inhibits NF-κB signaling more strongly due to its tighter binding to LUBAC.

### OTULIN Loss of Function Is Detected in Clinical TNBC Samples.

**Fig. 6.**
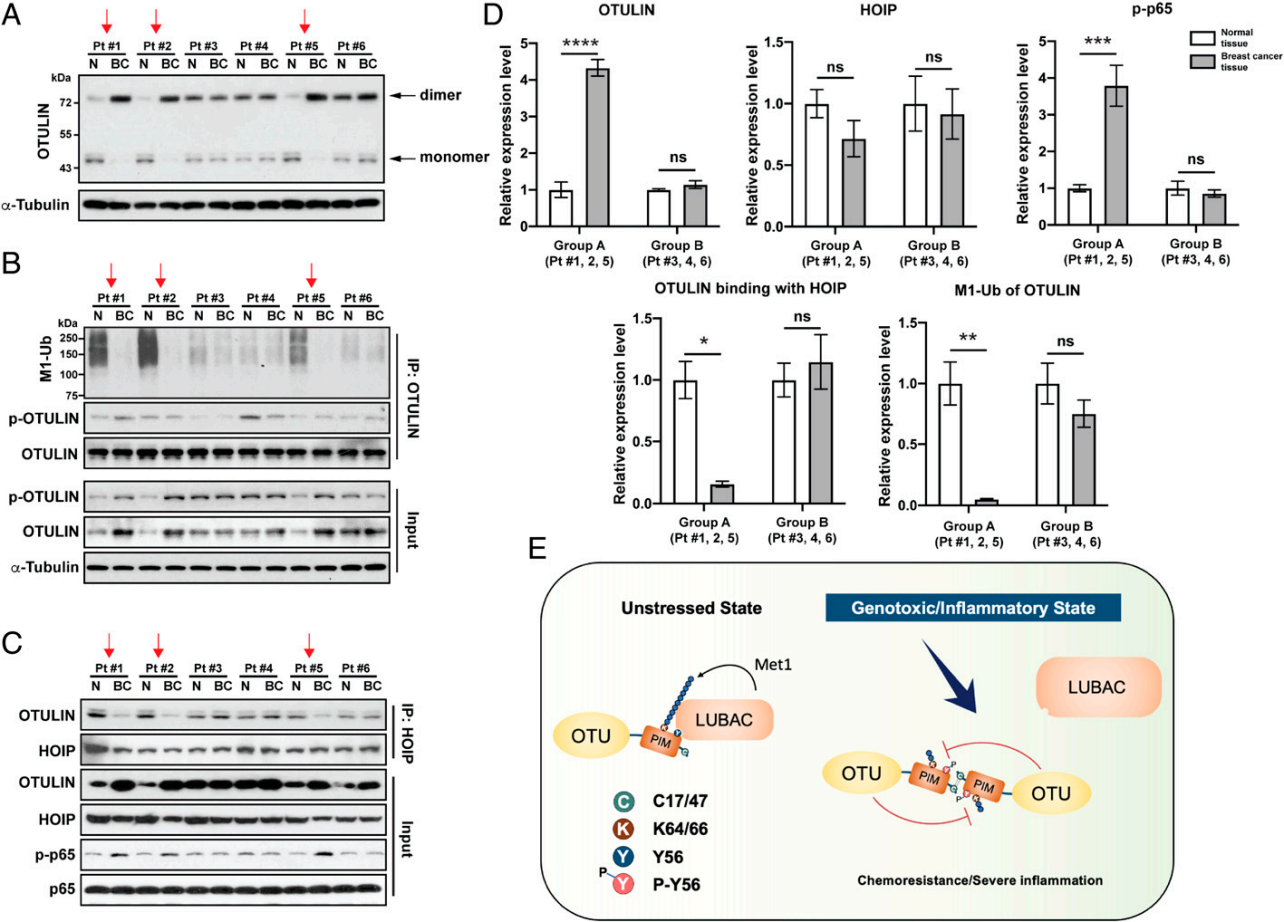
Analysis of OTULIN dimers, linear ubiquitination, and OTULIN-HOIP interaction in the breast cancer clinical samples. (*A*) Western blot analysis of OTULIN dimerization in breast cancer (BC) and adjacent nontumorous (N) tissue samples from six patients. Cell lysates were immunoblotted by indicated antibodies without DTT or 2-ME. (*B*) Linear ubiquitination and phosphorylation of immunoprecipitated OTULIN in breast cancer (BC) and adjacent nontumorous (N) tissue samples from six patients. Protein expression levels of total OTULIN and p-OTULIN are shown in the input. Cell lysates were immunoprecipitated with a low amount of Dynabeads protein A (10 μL) and anti-OTULIN antibody (2 μL) to make them saturated and immunoblotted with indicated antibodies. (*C*) Co-IP analysis of the interaction between HOIP and OTULIN in breast cancer (BC) and adjacent nontumorous (N) tissue samples from six patients. Protein expression levels of OTULIN, HOIP, and p-P65 are shown in the input. Cell lysates were immunoprecipitated with a low amount of Dynabeads protein A (10 μL) and anti-HOIP antibody (2 μL) to make them saturated and immunoblotted with indicated antibodies. (*D*) Quantification graph based on densitometry of the Western blot data from (*A*, *B*, and *C*). The data are presented as the mean ± SEM from three clinical samples. Statistical analysis was performed by a two-way ANOVA with Tukey’s correction for multiple comparisons. *P < 0.05; **P < 0.01; ***P < 0.001; ****P < 0.0001; ns, not significant. (*E*) Graphical abstract demonstrating the crucial roles OTULIN plays in NF-κB–mediated chemotherapy resistance and inflammation. Under unstressed conditions, OTULIN binds to HOIP, which limits the linear ubiquitination of the substrate molecules such as NEMO, governing a normal NF-κB activation. Under genotoxic or inflammatory conditions, OTULIN molecules dimerize, promoting Tyr56 phosphorylation and its self-deubiquitination, resulting in its dissociation from HOIP. Ultimately, OTULIN loss of function leads to an aberrant NF-κB activation and subsequent chemotherapy resistance or inflammatory symptoms. Pt, patient. Red arrows indicate the same individual samples that show loss of function of OTULIN (i.e., correlated loss of OTULIN–HOIP interaction and OTULIN deubiquitination).

Next, we determined whether the identified molecular events could be detected in clinical samples, including dimerization, linear ubiquitination, and LUBAC interaction with OTULIN. Three of the six human clinical samples displayed markedly increased expression levels of OTULIN in breast cancerous tissue (BC) compared with adjacent normal tissue (N), which correlated with the up-regulated OTULIN dimerization and linear deubiquitination and almost demolished the interaction with HOIP(Fig. 6 *A*–*C*, red arrows, group A). The increased OTULIN levels in these three samples were mostly in the dimerized forms with very little monomers; we observed a low degree of the OTULIN–HOIP interaction pattern in the cancerous tissue, which correlated with the low levels of monomers, indicating that only monomers can bind HOIP. Accordingly, these three samples displayed higher levels of NF-κB activation (p-p65/Ser536; Fig. 6*C*). On the contrary, in the remaining three specimens (group B), the relative relationship between the OTULIN protein levels and the OTULIN–HOIP interaction was constant without showing a significant difference between BC and N tissues, correlating with no significant change in NF-κB activation (Fig. 6*D*).

Bioinformatic analysis based on a published large proteomic database using a large cohort of primary breast tumor tissues (45) revealed that both OTULIN and LUBAC protein expression levels are altered significantly in the basal-like (a TNBC subtype) group (*SI Appendix*, Fig. S6*A*) compared with the other three groups, while CYLD is not significantly changed. Specifically, OTULIN protein expression is increased in 16 out of the 26 cases of the basal-like group (> 60%) at 2- to 8-fold: 6 cases at 2-fold, 6 cases at 4-fold, 2 cases at 6-fold, and 2 cases at 8-fold, while the increase of OTULIN expression in the other groups of human epidermal growth factor receptor 2-positive (HER2+) and luminal A and B are much milder (2-fold) among 30 to 36% of all cases. On the contrary, HOIP expression is mildly reduced in most of the 26 cases in the basal-like group as compared with other groups (*SI Appendix*, Fig. S6*B*). As in a recently published paper, transcriptomic analysis based on the TCGA-BRCA genomic dataset showed significantly higher mRNA levels of OTULIN in the BL subtype of breast cancer than that in other molecular subtypes (i.e., HER2+, luminal A, luminal B, and normal subtypes) (46). The heterogeneity of breast cancer biology is well recognized, which deeply challenges the drive for personalized treatment. The significant changes of OTULIN-LUBAC may reveal a path toward future personalized therapy.

## Discussion

It has been more than two decades since ubiquitin chains were discovered to modulate NF-κB activation, with the revelation that IκBα is modified with K48-linked ubiquitin chains in response to receptor activation, resulting in rapid proteasomal degradation (47–49). Since this discovery, K63-linked ubiquitin chains have been found to perform a nondegradative role in signal transduction and NF-κB activation by facilitating the activation of TAK1 (50, 51). Linear ubiquitin has been discussed in the literature for more than a decade (42). It is now clear that it harbors effective signaling properties to promote IKKβ phosphorylation by TAK1 (14, 35). Together with K48- and K63-linked chains, it plays crucial roles in NF-κB activation (6).

Applying multiple experimental approaches in cellular TNBC models, we demonstrate that OTULIN is linearly ubiquitinated by LUBAC under an unstressed condition that is required for stabilizing the interaction between OTULIN and HOIP, the core catalytic unit of LUBAC; this interaction counteracts LUBAC’s function by promoting OTULIN recruitment to the NF-κB substrates, such as NEMO. Furthermore, OTULIN blocks LUBAC-mediated genotoxic NF-κB activation by removing linear ubiquitin chains from NEMO (*SI Appendix*, Fig. S2). Meanwhile, we found that genotoxic stress induces OTULIN dimerization, which in turn mediates its own intermolecular deubiquitination. More important, OTULIN dimerization results in a disruption of the OTULIN-HOIP interaction, leading to genotoxic NF-κB activation and increased cancer cell survival (i.e., the main feature of chemoresistance). Our work discloses that the OTULIN–LUBAC interaction and the LUBAC-mediated OTULIN linear ubiquitination are the prerequisite events for OTULIN to exert its negative role in counteracting LUBAC-mediated genotoxic NF-κB activation. This conclusion is strongly supported by the human clinical evidence presented in Fig. 6*D*: three out of the six chemotherapy-resistant TNBC specimens display increased OTULIN expression in cancerous tissue compared with the adjacent normal breast tissue, which correlates with a nearly complete loss of OTULIN linear ubiquitination, significantly disrupted OTULIN–HOIP interaction (> 70%, *P* < 0.01), and increased NF-κB activation (Fig. 6*D*). Interestingly, these same three cancerous tissue specimens also showed a slightly decreased expression of HOIP protein. Remarkably, the reciprocally changed levels of OTULIN and HOIP revealed from these small sets of TNBC specimens were also reflected in large cohorts of breast cancer specimens; no significant change in CYLD protein expression was shown (*SI Appendix*, Fig. S6*A*). Together, these data indicate a prominent role of the OTULIN–LUBAC axis in regulating genotoxic NF-κB signaling: Increased OTULIN and lower LUBAC levels in TNBC patients are likely associated with poor prognosis, short survival, and increased chemotherapy resistance.

Note that our genotoxic cellular models did not recapitulate one aspect of the clinical samples on the high expression levels of OTULIN in the cancerous tissue that displayed nonfunctional OTULIN in NF-κB inhibition. In our cellular models under unstressed conditions, the majority of the overexpressed OTULIN stays as monomers and are associated with LUBAC, while they form dimers under acute genotoxic conditions via a mechanism of reactive oxygen species (ROS)-mediated Cys modification. Therefore, the inhibitory effect of OTULIN seems to depend on the functional OTULIN monomers rather than the dysfunctional OTULIN dimers. In the cancerous tissues that may undergo chronic stress over months or years, it is plausible to postulate that OTULIN is up-regulated as an adaptive stress response. However, upon sustained and perhaps high levels of oxidative stress during oncogenesis, both the ROS and the up-regulated OTULIN somehow facilitate its dimerization and the subsequent loss of function. Owing to the heterogeneity of clinical cancer tissues, OTULIN and LUBAC components may be dysregulated at additional multiple levels, involving transcriptional, posttranscriptional, and posttranslational mechanisms. Although these full events may not be easily recapitulated by the acute cellular model, the model we postulated for a chronic clinical condition does not contradict the overall findings we obtained from the cellular models and the concluded mechanisms.

It should be pointed out that our recently published work (46) illustrates OTULIN’s differential role in the positive regulation of the Wnt/β-catenin pathway, another crucial signaling mechanism underlying chemotherapy resistance upon overactivation. Using afore-described genotoxic cellular models, we demonstrate that unlike in the case of the NF-κB complex, in which the OTULIN–HOIP interaction is imperative for OTULIN recruitment to NEMO, the phosphorylated state of OTULIN (Tyr56) but not the OTULIN–HOIP interaction is required for OTULIN recruitment to β-catenin. Interestingly, genotoxic stress induces OTULIN phosphorylation on Tyr56 (p-OTULIN), leading to OTULIN disassociation from the HOIP/LUBAC complex (Fig. 2*E*); the released p-OTULIN can bind and deubiquitinate the linear chains of β-catenin. Meanwhile, the p-OTULIN-β-catenin complex somehow also reduces the K48 chains, thereby stabilizing β-catenin from proteasomal degradation. Therefore, OTULIN is involved in at least two major signaling pathways underlying chemotherapy response, via distinct roles. The relative interplay between these two pathways for their contributions and balance warrants further investigation.

Hyperphosphorylation on the Tyr56 has previously been identified as a major molecular mechanism leading to the disruption of OTULIN from the LUBAC complex (22, 23), presumably because this residue is in a most critical position regarding the N-terminal domain-binding motif of OTULIN to HOIP’s PUB domain(22). In this work, we discover that genotoxic stress induces OTULIN dimerization (Fig. 4*F*), which is also likely mediated by mechanisms through oxidative stress (H_2_O_2_; Fig. 5*B*). In both of our genotoxic and oxidative stress models, OTULIN dimerization and its phosphorylation on the Tyr56 site result in the dissociation of OTULIN from the HOIP/LUBAC complex.

Protein self-association to form dimers and higher-order oligomers is quite a common phenomenon that is crucial for the regulation of proteins such as enzymes, receptors, ion channels, and transcription factors (52). For example, the homodimeric protein NEMO linked by disulfide bridges is required for TNFα-induced NF-κB activation (53). We found that OTULIN dimers are undetectable under normal protein isolation conditions but only appear with a DSS cross-linker (Fig. 4*G*). Furthermore, the N terminus of OTULIN is required and sufficient for its dimerization. Interestingly, the selective Cys-mutated OTULIN failed in both dimerization and Tyr56 phosphorylation under these stressed conditions (Fig. 5*G*), indicating that dimerization is required for the critical Tyr56 residue to be phosphorylated. These actions may be explained by potential structural conformational changes. The resolved crystal structure shows the Tyr56 location in a close position to the critical catalytic domain (22, 23), which may be inaccessible to any kinase (s) under unstressed conditions and is therefore unphosphorylated. We further speculate that OTULIN dimerization through covalent bonds formed by Cys47 (and or Cys17) release Tyr56 from the PUB pocket of HOIP, leading to the exposed Tyr56 residue to be phosphorylated by the kinase under a genotoxic condition (i.e., ABL1, the oncogenic tyrosine kinase recently identified in our prior work) (46). ABL1 has been frequently reported to be activated upon oxidative stress. Therefore, targeting oxidative stress represents an invariant therapeutic strategy, probably not only for combating chemotherapy resistance (e.g., via preventing OTULIN dimerization and Tyr56 phosphorylation) but also for almost all human diseases.

The other finding in this work is that OTULIN is linearly ubiquitinated on K64/66 sites by LUBAC–HOIP, which is required for OTULIN to stably interact with the LUBAC complex through direct binding to HOIP. OTULIN was also recently reported to be K63-ubiquitinated by tripartite motif-containing protein 32 (TRIM32) at the same K64/66 residues under TNFα stimulation (54). Paradoxically, in this published work, the K63-ubiquitinated OTULIN loses binding to LUBAC, resulting in overactivated NF-κB. In theory, both K63 and linear ubiquitination machinery can compete for the K64/66 sites, depending on the specific upstream signals. Here, we present compelling evidence collected from multiple cellular models, including both 293T and MDA-MB-231 cells, demonstrating that OTULIN linear ubiquitination assembled by LUBAC in resting cells is required for stabilizing OTULIN–LUBAC interaction and limiting genotoxic NF-κB activation. Genotoxic stress primarily induces dissociated OTULIN–LUBAC via dimerization and Tyr56 phosphorylation. We also observed OTULIN self-deubiquitination, which is predicted to be dependent on dimer formation. Although the deubiquitinated OTULIN dimers may still harbor catalytic DUB activity, the loss of contact between them and LUBAC leads to a loss of function of OTULIN.

Given the paramount importance of the NF-κB pathway not only in cancer development and chemotherapy resistance (55–57) but also in receptor-mediated innate and adaptive immune responses (e.g., TNFα, interleukin-1, and interferons), our mechanistic findings based on studying genotoxic response will likely be generalized to canonical NF-κB signaling. Indeed, we reproduced all key findings in the TNFα-induced stress response (Fig. 6 *A*–*C*). Negative feedback mechanisms are crucial to limit and fine-tune overactivated NF-κB signaling (7). DUBs have emerged as an important class of enzyme molecules playing an important part in this process. Findings from our work fill a knowledge gap in terms of ubiquitin assembling and disassembling.

In summary, we conclude that OTULIN plays an indispensable role in counteracting LUBAC-mediated genotoxic NF-κB activation; loss of function of OTULIN contributes to the development of a chemotherapy-resistant phenotype at least in TNBC culture and clinical models as well as under inflammatory stimulation by TNFα. We identified multiple molecular events on OTULIN that commonly occur upon genotoxic and inflammatory stresses (summarized in Fig. 6*E*): 1) OTULIN linear ubiquitination on K64/66 by LUBAC-HOIP is crucial for maintaining its physical interaction with the LUBAC complex through direct binding with the PUB domain of HOIP; 2) in genotoxic or proinflammatory conditions, OTULIN undergoes dimerization and self-deubiquitination on the K64/66 sites, allowing Tyr56 phosphorylation to occur; subsequently, these events result in OTULIN’s dissociation from HOIP and the LUBAC complex, leading to increased NEMO linear ubiquitination and overactivated NF-κB signaling. Our study not only reveals a mechanical regulation of LUBAC signaling but also identifies the OTULIN–LUBAC interaction as a promising target for attenuating chemotherapy resistance via NF-κB signaling. Effective and practical strategies of stabilizing OTULIN linear ubiquitination and the OTULIN–LUBAC interaction require a fuller understanding of OTULIN-mediated molecular mechanisms. New OTULIN substrates may continue to be identified. There are more recent reviews mentioning OTULIN’s distinct roles that are dependent on and independent of its DUB activity, as well as its potential cell type–specific functions (27), which need to be validated in the future.

Finally, TNFα-induced NF-κB overactivation is arguably the most crucial signaling pathway underlying a myriad of autoinflammatory and autoimmune conditions, including the current SARS-CoV-2 pandemic (i.e., cytokine storms) (58). Bioinformatic analysis for evidence of a potential role of OTULIN from a limited public database of COVID-19 patients suggests a potentially negative correlation between the increased OTULIN levels and patients’ hyperinflammatory response (59–61). Furthermore, the OTULIN–LUBAC axis has been previously reported to be crucial in the host defense against other pathogens (62, 63). Taken together, increasing compelling evidence supports a general treatment strategy of targeting the NF-κB pathway as a potential treatment for critical-stage COVID-19 patients (64).

## Materials and Methods

### Reagents and Antibodies.

CPT, CBP, Etop, Dox, and gliotoxin were purchased from Sigma-Aldrich. Human and mouse recombinant TNFα was from CalBiochem and prepared in phosphate-buffered saline containing 0.1% bovine serum albumin fraction V (Sigma-Aldrich) to a final stock concentration of 10 μg/mL IgGs against c-Myc (9E10), GFP (B2), IκB-α (C-21), GST (B-14), p65 (F-6), ubiquitin (P4D1), and CYLD (H6) purchased from Santa Cruz Biotechnology. IgGs against HA (C29F4), His (D3I10), p-p65 (Ser536) (93H1), p-IκBα (Ser32) (14D4), K63-Ub (D7A11), K48-Ub (D9D5), cleaved caspase-3 (Asp175) (5A1E), RNF31 (E6M5B), OTULIN (14127s), and α-tubulin (DM1A) were purchased from Cell Signaling Technology. Anti-HOIL-1 antibody (2E2) and anti-linear ubiquitin antibody (LUB9) were obtained from Millipore Sigma. Anti-SHARPIN antibody (ab197853) was purchased from ABCAM. *N*-ethylmaleimide (catalog no. 23030) and IAM (catalog no. A39271) were purchased from Thermo Scientific.

### Cell Models.

MEFs, HEK293T, and MDA-MB-231 cells were cultured in DMEM supplemented with 10% fetal bovine serum and penicillin/streptomycin in a 5% CO_2_ humidified incubator. HOIL-deficient, cpdm, and WT MEFs were kind gifts from Dr. K. Iwai (Osaka University). Multiple stable clones of HEK293T_HOIP-KO (two clones), HEK293T_OTULIN-KO (three clones), and MDA-MB-231_OTULIN-KO (three clones) cells were generated using the CRISPR-Cas9 system developed by Dr. Feng Zhang (Massachusetts Institute of Technology) as described in the Supporting Information (SI). The sequences of Sg1 HOIP, Sg2 HOIP, and Sg1 OTULIN are good for generating completely KO cells. Representative data of all clones are shown in all the figures.

### Clinical Sample and Data Collection.

Immunoblotting and immunoprecipitation analysis of OTULIN was performed in breast cancer patient tumor samples and adjacent normal breast tissue samples. Specimens were collected immediately after surgery in RPMI medium containing penicillin:streptomycin or were snap-frozen and transported to the laboratory on ice. The patient tumors and normal tissues were deidentified and collected under a University of Tennessee Health Sciences Center institutional review board–approved protocol (14–03113XP).

### Electrophoretic Mobility Shift Assay.

The Igκ-κB oligonucleotide probe and electrophoretic mobility shift assay wee performed as described previously (14). The Oct-1 site oligo was purchased from Promega as a control.

## Supplementary Material

Supplementary File

## Data Availability

All processed data, including raw data, are included in the article or can be obtained from the first and corresponding authors.
