# Supplementary material for: Reciprocal interplay between OTULIN–LUBAC determines genotoxic and inflammatory NF-κB signal responses
Source: Proc Natl Acad Sci U S A. 2022 Aug 8;119(33):e2123097119. doi: 10.1073/pnas.2123097119 (PMC9388121; doi:10.1073/pnas.2123097119)
Supplement: Supplementary File [file pnas.2123097119.sapp.pdf]

PNAS

Supplementary Information for
Reciprocal interplay between OTULIN-LUBAC determines genotoxic and inflammatory NF-κB signal responses

Mingqi Li[1*], Ling Li[2], Sarah Asemota[3], David Kakhniashvili[4], Ramesh Narayanan[3], Xusheng Wang[2], Francesca-Fang Liao[1*].

Francesca-Fang Liao, Ph.D.
e-mail: fliao@uthsc.edu

Mingqi Li, M. S.
e-mail: Mli59@uthsc.edu

This PDF file includes:

Methods
Figures S1 to S6
Table S1 and S2
SI References (1, 2)

SI Materials and Methods

Target guide sequence cloning with CRISPR/Cas9. Different single guide RNA sequences targeting OTULIN or HOIP were acquired from Genome-scale CRISPR Knock-OUT v2 libraries [1]. Each pair of Oligos (100 μM) were annealed in the mixture with T4 ligation buffer (B0202S; BioLabs) and ddH2O at 37°C for 30 min followed by 95°C for 5 min and cool down to room temperature (RT) naturally. A total of 5 μg of LentiCRISPRv2 was digested by BsmBI in FastDigest buffer for 30 min at 37°C. The annealed oligos above were ligated with agarose gel-purified vector using QIAquick Gel Extraction Kit (cat# 28704; QIAGEN). The Lenti-sgOTULIN was subsequently generated by transformed competent DH5α, which was selected by ampicillin.

Lentivirus packaging and transduction of cell lines and generation of stable cell line. Lentiviral plasmid (lenti-CRISPR-ver2.0-sg-OTULIN), Vsv-g, pMDL-g, and RSV-REV were co-transfected into HEK293T cells for viral packaging. After 72 h, culture media was harvested and centrifuged at 1800 rpm, 5 min at RT to acquire cell debris-free supernatant. The filtered media by 0.45 μm filter mixed with 6 μg/ml polybrene was added to target cells with about 70% confluence. Screen stable single clones by antibiotic puromycin (2 μg/ml) selection.

Cell viability assay. After transfected with plasmids, cells were seeded into 96-well plates (5 x 10^3/well) in triplicate. After doxorubicin treatment for 48 h, cells were incubated with CCK8 reagents (10 μl/well; Dojindo) for 2 h and plates were measured at OD450 in a microplate reader.

Luciferase assay. Cells for NF-κB signaling reporter assay were co-transfected with 0.5 μg pGL4.32 (Luc2p/NF-κB–RE/Hygro), 10 ng pGL4.74 (hRLuc/TK) (Promega), and the indicated plasmids per well of 24-well plate in triplicate. After 24 h, cells were treated with compounds as indicated in the figure and the luciferase activity was detected by a Promega GloMax 20/20 luminometer, using the Dual-Luciferase Reporter (DLR) Assay System (cat# E1960; Promega). Renilla luciferase activity was measured for normalization of transfection efficiency.

DSS crosslinking assay. HEK293T cells were treated with 2 μM of doxorubicin or DMSO for 2 hours. ~1.2×10^6 cells were then washed three times and collected with 500 μl cold PBS (20 mM sodium phosphate, 0.15M NaCl; pH 8). Crosslinker disuccinimidyl suberate (DSS) solution (ThermoFisher, 21655) was added to a final concentration of 1 mM for incubation for 30 min at RT. The reaction was stopped by the quench solution with a final concentration of 10 mM Tris-HCl (pH 7.5) for 15 min at RT. Finally, cells were obtained by 200 g centrifugation for OTULIN dimerization analysis by WB.

Immunoblotting and Immunoprecipitation. For immunoblotting, cells were lysed with the RIPA buffer (Thermo Scientific) containing protease inhibitor cocktail (Roche) and phosphatase inhibitor cocktail (Roche). After centrifuging the cell lysates at 18×10^3 g for 10 min, protein concentration was determined by Bradford protein assay (Bio-Rad), and the same amounts of lysates (30 μg) were dissolved in 2 X sample buffer (125 mM Tris-HCl, 4% SDS, 20% glycerol, 10% 2-mercaptoethanol (2-ME), a pinch of bromophenol blue) and boiled for 10 min at 95°C. Alternatively, lysates were dissolved in 2 X sample buffer without 2-ME to detect the dimerization of OTULIN. Samples were loaded for SDS-PAGE and immunoblotted with the appropriate antibodies. The proteins of interest were visualized by chemiluminescent substrate.

For co-immunoprecipitation (co-IP) assays, the whole cells of 60 mm dish were collected in 1.5 ml Eppendorf tube and lysed by homemade IP lysis buffer (20 mM Tris (pH 7.0), 150 mM NaCl, 1 mM EDTA, 1 mM EGTA, 0.5% NP-40, 2 mM DTT, 0.5 mM PMSF, 20 mM β-glycerol phosphate, 1 mM sodium orthovanadate, 1 μg/ml leupeptin, 1 μg/ml aprotinin, 10 mM *p*-nitrophenyl phosphate, and 10 mM sodium fluoride) with protease inhibitor cocktail. After the centrifugation, 5% supernatant lysates were taken as input for future use. Dynabeads Protein A (20 μl) was prepared by washing three times with IP lysis buffer and then incubated with 1 μg primary antibody and the left cell lysates at 4°C overnight. The protein-antibody-bead conjugates were isolated by magnetic rack, followed by five washes with IP lysis buffer.

For ubiquitin detection of immunoprecipitated protein, cells were boiled for 30 min at 95°C in IP lysis buffer containing 1% SDS to break noncovalent connections. After heating, the cell lysates were centrifuged at 18×10^3 g for 10 min at 4°C. The resultant supernatant was diluted with IP lysis buffer to 0.1% SDS (final volume 1 ml) and incubated with primary antibody (1 μg) and Dynabeads Protein A (20 μl) at 4°C overnight.

Immunoprecipitation to detect DCP-Bio1-labeled OTULIN. For analysis of protein sulfenylation (-SOH), cells were lysed with RIPA lysis buffer containing 500 μM DCP-Bio1 (Kerafast, Boston, MA) and protease inhibitor cocktails on ice for 1h. After the centrifugation and quantification, 30 μg protein was used for input; 300 μg of each sample was preincubated with 20 μl Dynabeads-protein A magnetic beads preequilibrated in lysis buffer (no antibody) for 2 h at 4°C, and beads were isolated by magnetic rack and discarded to exclude the nonspecific protein-beads binding. In addition, a fresh aliquot (20 μl) of beads was preincubated with 3 μl of anti-HA

antibody for 2 h at 4°C. The antibody-crosslinked beads were collected and incubated with preprocessed lysates overnight at 4°C. The next day, supernatants were removed, and beads were washed three times with cold RIPA buffer. HA-OTULIN protein was eluted with 25 μL SDS sample buffer by boiling at 95°C for 10 min. Total supernatants of eluted protein and SDS sample buffer-boiled Input were loaded into 4-20% gradient polyacrylamide gels and separated by SDS-PAGE. The subsequent procedures were PVDF membrane transfer, 5% milk blocking, and incubation with HRP-conjugated anti-biotin antibody (1:1000) or anti-HA antibody (1:1000) overnight at 4°C. For the latter one, additional incubation with an HRP-conjugated secondary IgG antibody for 2 h at 24°C was needed. PVDF membrane was washed with TBST buffer for 30 min, replacing fresh buffer every 10 min. Finally, protein of interest was visualized by chemiluminescent approach.

Expression and purification of glutathione-s-transferase (GST) or histidine (His)-tagged fusion proteins. pGEX-5X.1-GST-PIM, pGEX-5X.1-GST-PIM-Ub4, pGEX-4T-2-GST-PUB_WT, pGEX-4T-2-GST-PUB_D117A, pGEX-4T-2-GST-PUB_E127A, pGEX-4T-2-GST-PUB_D117A/E127A, pET28a-His-PUB, pET28a-His-PIM-Ub4, and pET28a-His-Ub4 constructs were expressed in BL21 (DE3) cells. Cells with GST or His constructs were grown at 37 ° C in 250 ml LB medium with 100 μg/ml ampicillin or 50 μg/ml kanamycin, respectively, to an OD600 of 0.4-0.6. The culture was further incubated with IPTG to a final concentration of 0.1 mM for 2.5 h. Cells were collected at 5000 g for 15 min at RT. Pellet resuspending in 5 ml ice-cold PBS with cocktail proteinase inhibitor was sonicated for lysis. Triton X-100 was added to a final concentration of 1% to avoid the association of fusion protein with bacterial proteins. To remove insoluble material and cell debris, the mixture was centrifuged for 5 min at 10,000 g at 4 ° C. GST- or His-containing supernatant was added with 1 ml of 50 % slurry of glutathione-agarose beads or Ni-NTA agarose beads, respectively, and mix for 2 h at 4 ° C. After centrifugation at 500 g for 10 sec, beads were collected and washed by PBS for elution, in which glutathione-agarose beads or Ni-NTA agarose beads were mixed with reduced glutathione or imidazole, respectively, in 500 μl 50 mM Tris (pH 8.0) elution buffer.

In vitro pulldown assay. Glutathione-agarose beads or Ni-NTA agarose beads and 2 µg of each purified fusion protein of interest were added into one 1.5 ml Eppendorf tube and incubated for 2 h at 4 °C in 500 µl IP buffer with cocktail proteinase inhibitor. Beads were washed three times by IP buffer to remove non-bound protein before resuspended in 20 µl 2x SDS loading buffer for WB.

Mass spectrometry. For in-gel digestion, OTULIN gel band was performed overnight at 37°C using In-Gel Tryptic Digestion kit (cat# 89871x, Thermo Scientific) according to the manufacturer's protocol including optional steps for reduction and alkylation (carbamidomethylation). The generated peptide mixture (25 μl) was separated from the gel piece and vacuum dried.

For on-beads digestion, beads with IP OTULIN were transferred into 70 μl of digestion buffer (DB, 100 mM ammonium bicarbonate) and incubated with 4 μl of LysC/Trypsin mixture (cat# A41007, Thermo Scientific) dissolved at 0.1 μg/μl in DB. Trypsin/Lys-C digestion was performed overnight at 37°C and 1150 rpm using Eppendorf ThermoMixer F1.5. The peptide digests were collected, and the beads were extracted with 100 μl of 50% acetonitrile/0.5% TFA at 37°C for 10min; the extracts were combined with corresponding digests, desalted using Pierce C-18 spin tips (cat# 84850, Thermo Scientific) according to the manufacturer's protocol, and vacuum dried.

For LC-MS/MS assay, the dried peptide sample was re-dissolved in 30 μl of loading buffer (3% acetonitrile, 0.1% TFA), and 5 μl was analyzed using LC-MS-MS method with 160 min LC gradient for peptide/protein identification and mapping of specified PTM sites. Raw MS data were acquired on an Orbitrap Fusion Lumos mass spectrometer (Thermo Fisher) as described previously (1).

The analysis of the acquired raw MS data was performed within a mass informatics platform Proteome Discoverer 2.2 (Thermo Fisher) as described previously (1).

Statistical analysis.
The results were presented as mean ± SEM. Statistical analysis has been performed using Prism GraphPad 9 software. The number of independent experiments, experimental repeats, biological samples are indicated in figure legends. Multiple comparisons were made using one-way or two-way ANOVA as shown in the figure legends. $p < 0.05$ is considered significant, indicated as $*p < 0.05$, $**p < 0.01$, $***p < 0.001$, and $****p < 0.0001$.

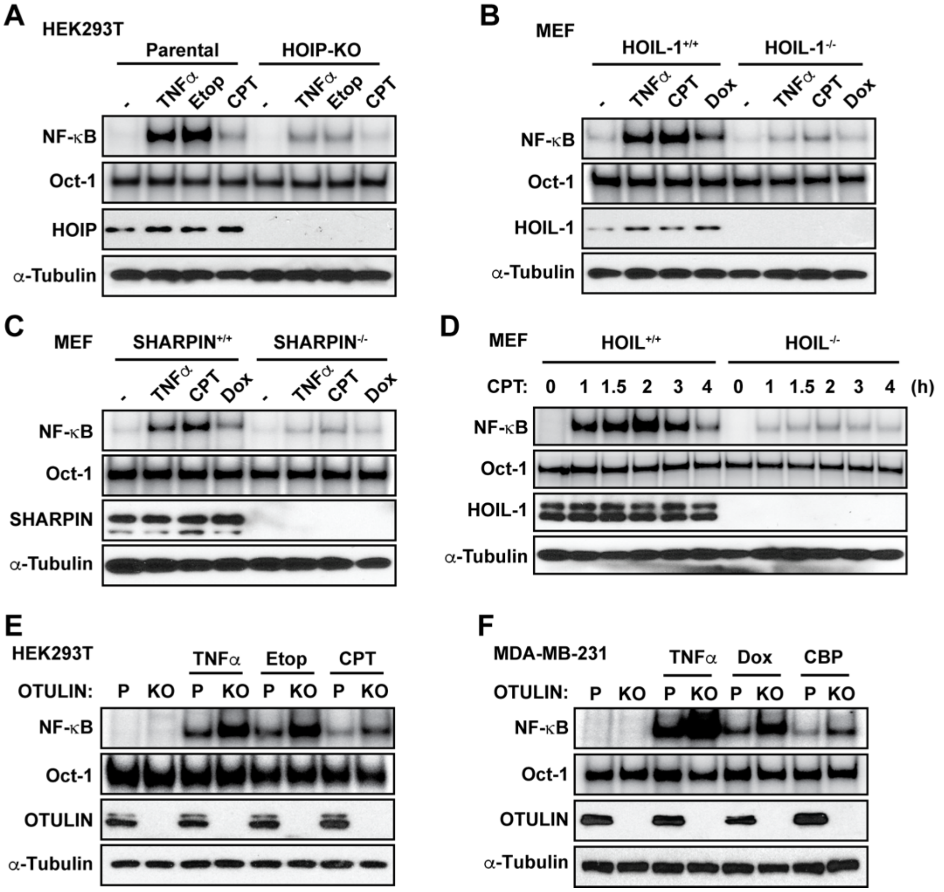

Fig. S1. LUBAC (HOIP) and OTULIN plays opposing roles in mediating genotoxic NF-κB activation. (*A*) Gel shift analysis of NF-κB activation by using Igκ probe and Western blot analysis of HOIP in parental and HOIP-KO 293T cells treated with TNFα (10 ng/ml, 15 min), Etop (10 μM, 2 h), or CPT (10 μM, 2 h). (*B*) Gel shift analysis of NF-κB activation by using Igκ probe and Western blot analysis of indicated proteins in HOIL-1$^{+/+}$ and HOIL-1$^{-/-}$ MEFs treated with TNFα (10 ng/ml, 15 min), CPT (10 μM, 2 h), or Dox (2 μg/ml, 2 h). (*C*) SHARPIN$^{+/+}$ and SHARPIN$^{-/-}$ MEFs were treated and analyzed as in (B). (*D*) HOIL-1$^{+/+}$ and HOIL-1$^{-/-}$ MEFs were treated with CPT (10 μM) for indicated time points, and then analyzed by gel shift and Western blot. (*E*) Parental and OTULIN-KO 293T cells were treated with TNFα (10 ng/ml, 15 min), Etop (10 μM, 2 h), or CPT (10 μM, 2 h), and then analyzed by gel shift and Western blot. (*F*) Parental and OTULIN-KO 293T cells were treated with TNFα (10 ng/ml, 15 min), Dox (2 μg/ml, 2 h), or CBP (10 μg/ml, 2 h), and then analyzed by gel shift and Western blot.

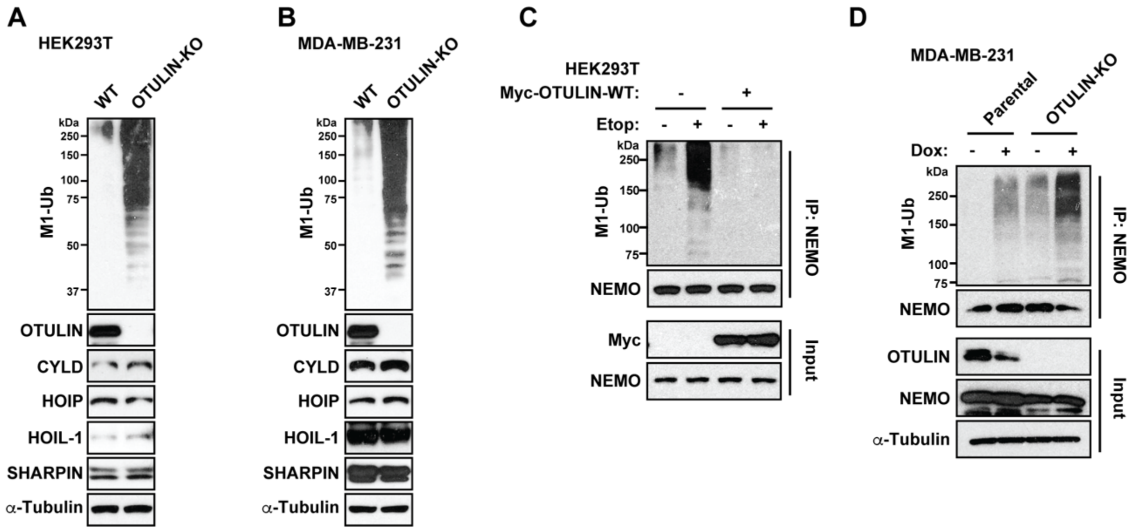

Fig. S2. Linear ubiquitinated NEMO levels are increased upon OTULIN-null condition. (*A* and *B*) Western blot analysis of parental and OTULIN-KO clones in 293T and MDA-MB-231 cells using the antibodies as indicated. (*C*) Linear ubiquitination of immunoprecipitated NEMO. HEK293T cells were transfected with or without Myc-OTULIN for 48 h, and then treated with Etop (10 μM, 2 h) or left untreated (-). Cell lysates were immunoprecipitated with anti-NEMO antibody and immunoblotted with indicated antibodies. (*D*) Linear ubiquitination of Immunoprecipitated NEMO. Parental and OTULIN-KO MDA-MB-231 cells were treated with or without Dox (2 μg/ml, 2 h). Cell lysates were immunoprecipitated with anti-NEMO antibody and immunoblotted with indicated antibodies.

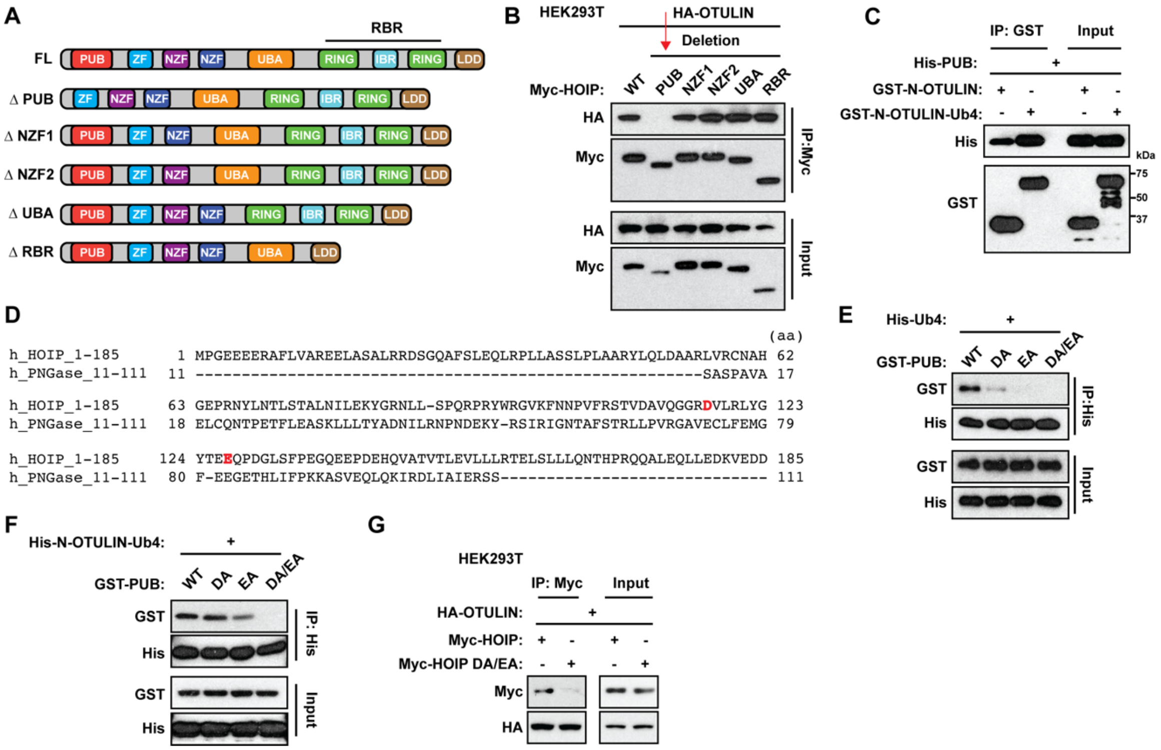

Fig. S3. N-terminus of OTULIN interacts with HOIP-PUB which is facilitated by linear ubiquitin chains on OTULIN. (*A*) Diagram of FL HOIP and its indicated domain-deleted mutants: HOIP-PUB deletion (52-162aa), HOIP-NZF1 deletion (350-379aa), HOIP-NZF2 deletion (399-438aa), HOIP-UBA deletion (564-615aa), HOIP-RBR deletion (699-1072aa). (*B*) Co-IP analysis of the interaction between HA-OTULIN and Myc-HOIP WT, PUB-, NZF1-, NZF2-, UBA-, or RBR-deleted truncated mutant in HEK293T cells. Cells were co-transfected with HA-OTULIN WT and indicated Myc-HOIP constructs. After 48 h, cell lysates were immunoprecipitated with anti-Myc antibody and immunoblotted with antibodies as indicated. (*C*) *In vitro* pulldown assay of His-PUB (1-185aa) by GST-N-terminal OTULIN (1-80aa) or GST-N-terminal OTULIN fused with tetra linear ubiquitin chains (GST-N-OTULIN-Ub4). (*D*) Structure-based sequence alignment of HOIP and PNGase PUB domains. Red labeled letters in HOIP represent the corresponding conserved residues to Glu73 and Glu84 of PNGase. (*E* and *F*) *In vitro* pulldown assay of GST-PUB (1-185aa) domain WT, D117A, E127A, or D117A/E127A mutant by His-Ub4 and His-N-OTULIN-Ub4, respectively. (*G*) Co-IP analysis of the interaction between HA-OTULIN and Myc-HOIP in HEK293T cells. Cells were co-transfected with HA-OTULIN and Myc-HOIP WT or its D117A/E127A mutant. After 48 h, cell lysates were immunoprecipitated with anti-Myc antibody and immunoblotted with anti-Myc and anti-HA antibodies.

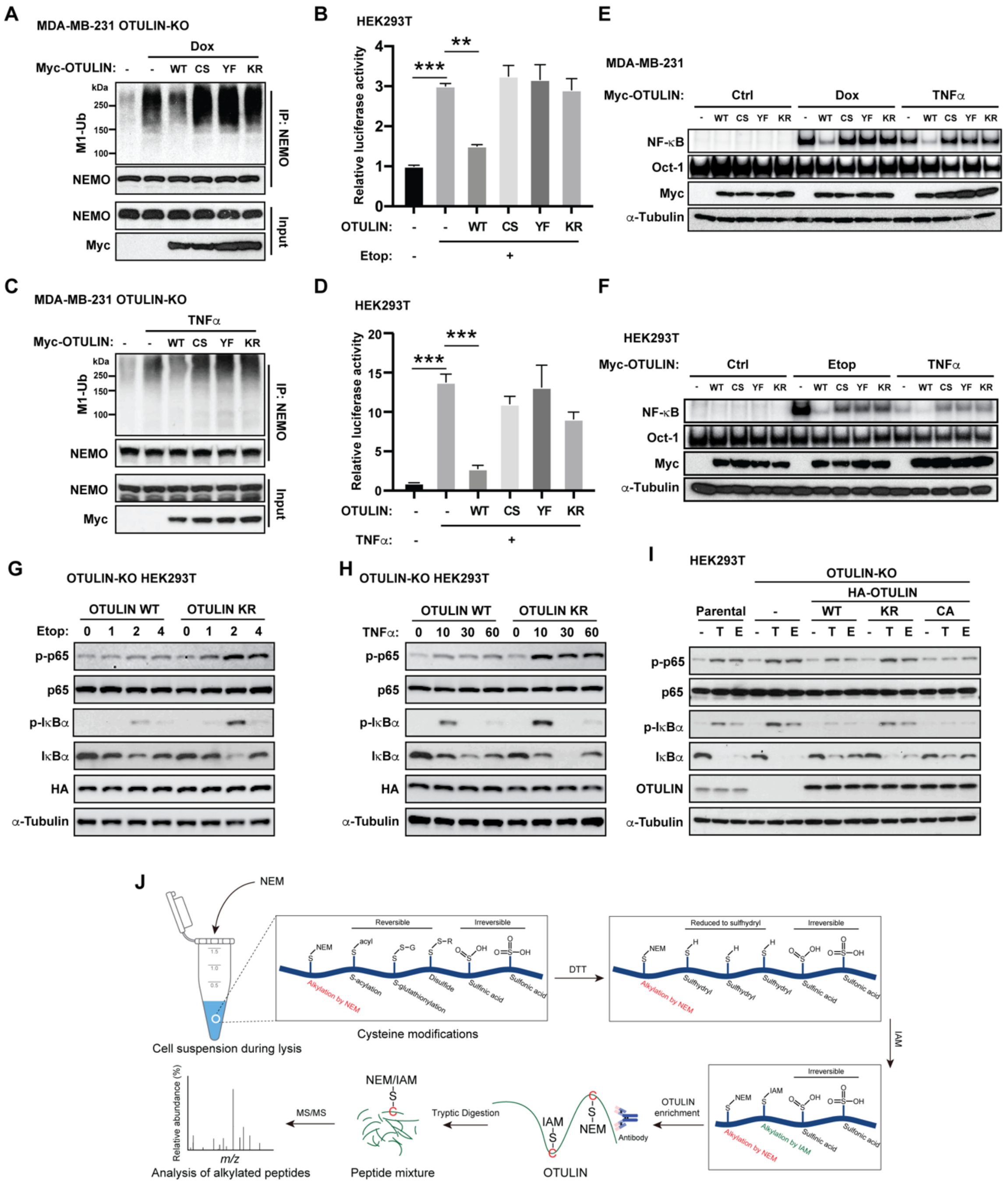

Fig. S4. Effects of OTULIN loss of function mutants on the status of linear ubiquitination of NEMO and NF-κB activation. (*A* and *C*) Linear ubiquitination of immunoprecipitated NEMO in OTULIN-KO MDA-MB-231 cells reconstituted with vehicle, Myc-OTULIN WT, C129S, Y56F, or K64/66R mutant for 48 h, and then treated with Dox (2 μg/ml, 2 h) in (*A*) or TNFα (10 ng/ml, 15 min) in (*C*). Cell lysates were immunoprecipitated with anti-NEMO antibody and immunoblotted with indicated antibodies. (*B* and *D*) HEK293T cells were co-transfected with κB-Fluc/hRluc-TK reporter constructs and HA-OTULIN WT, C129S, Y56F, or K64/66R mutant. After 24 h, cells were treated with or without Etop (10 μM, 6 h) in (*B*) or TNFα (10 ng/ml, 4 h) in (*D*), and then cell extracts were prepared to determine luciferase activity. The activities of firefly and *Renilla* luciferases were

evaluated sequentially from a single sample in the DLR Assay. Results were plotted as firefly normalized to *Renilla* luciferase activity. The relative luciferase activities compared with the control (lane 1) are shown as mean ± SEM from the experiments performed in triplicate. One-way ANOVA followed by Tukey's post-hoc test was used to determine statistical significance for multiple comparisons. $^{**}p < 0.01$; $^{***}p < 0.001$. (*E*) Gel shift analysis of NF-κB activation using Igκ probe and Western blot analysis of Myc in MDA-MB-231 cells transfected with Myc-OTULIN WT, C129S, Y56F, or K64/66R for 48 h, and then treated with Dox (2 μg/ml, 2 h) or TNFα (10 ng/ml, 15 min). (*F*) Gel shift analysis of NF-κB activation using Igκ probe and Western blot analysis of Myc in HEK293T cells transfected with Myc-OTULIN WT, C129S, Y56F, or K64/66R for 48 h, and then treated with Etop (10 μM, 2 h) or TNFα (10 ng/ml, 15 min). (*G*) Western blot analysis of kinetic NF-κB activation in OTULIN-KO HEK293T cells reconstituted with HA-OTULIN WT or K64/66R mutant for 48 h and then treated with Etop (10 μM) for indicated time points. Cell lysates were immunoblotted with indicated antibodies. (*H*) Western blot analysis of kinetic NF-κB activation in OTULIN-KO HEK293T cells reconstituted with HA-OTULIN WT or K64/66R mutant for 48 h and then treated with TNFα (10 ng/ml) for indicated time points. Cell lysates were immunoblotted with indicated antibodies. (*I*) Western blot analysis of NF-κB activation in parental, OTULIN-KO, and OTULIN-reconstituted HEK293T cells. OTULIN-KO HEK293T cells were reconstituted with HA-OTULIN WT, K64/66R, or C17/47A mutant. After 48 h, cells were treated with or without Etop (10 μM, 2 h) or TNFα (10 ng/ml, 15 min). Cell lysates were immunoblotted with antibodies as indicated. (*J*) Workflow of the identification of alkylated peptides of enriched OTULIN. During cell lysis with HEPES lysis buffer (cat# J63867, Thermo Scientific) containing protease inhibitor cocktails, NEM (5 mM, 60 min, RT) was firstly used to label reduced cysteine, then DTT (10 mM, 45 min, RT) was used to reduce the reversible cysteine, and then IAM (40 mM, 20 min, RT) was used to label the just reduced cysteines (i.e., the levels of IAM-labeled cysteines indicate the cysteine oxidation). Cell lysates were then acquired by centrifuge and applied for OTULIN IP as described in the co-IP section. Enriched OTULIN was used for the analysis of alkylated peptides by LC/MS/MS.

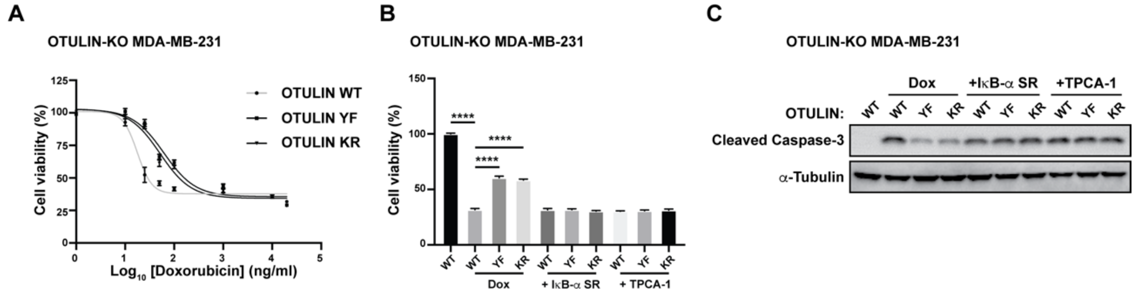

Fig. S5. Effects of OTULIN loss of function mutants on cancer cell survival via dysregulated genotoxic NF-κB signaling. (*A*) Representative data from three independent experiments with triplicate samples on the cell viability analyzed by the CCK-8 assay in OTULIN-reconstituted MDA-MB-231 cells treated with Dox for 48 h. Dox IC_{50} is 17.76 ng/ml, 61.72 ng/ml, and 51.67 ng/ml in HA-OTULIN WT-, Y56F-, or K64/66R-reconstituted cells, respectively. (*B*) Cell viability analyzed by CCK-8 assay in OTULIN-KO MDA-MB-231 cells reconstituted with HA-OTULIN WT, Y56F, or K64/66R mutant. Cells were co-transfected with IκBα-SR or pretreated with TPCA-1 (1 μM) for 24 h, and then treated with Dox (100 ng/ml, 48 h). The data are presented as the mean ± SEM from 5 biological replicates. Statistical analysis was performed by one-way ANOVA with Tukey's correction for multiple comparisons. ****$p < 0.0001$. (*C*) Western blot analysis of cleaved caspase-3 in OTULIN-KO MDA-MB-231 cells treated as in (*B*). Cell lysates were immunoblotted with anti-cleaved caspase-3 antibody.

A

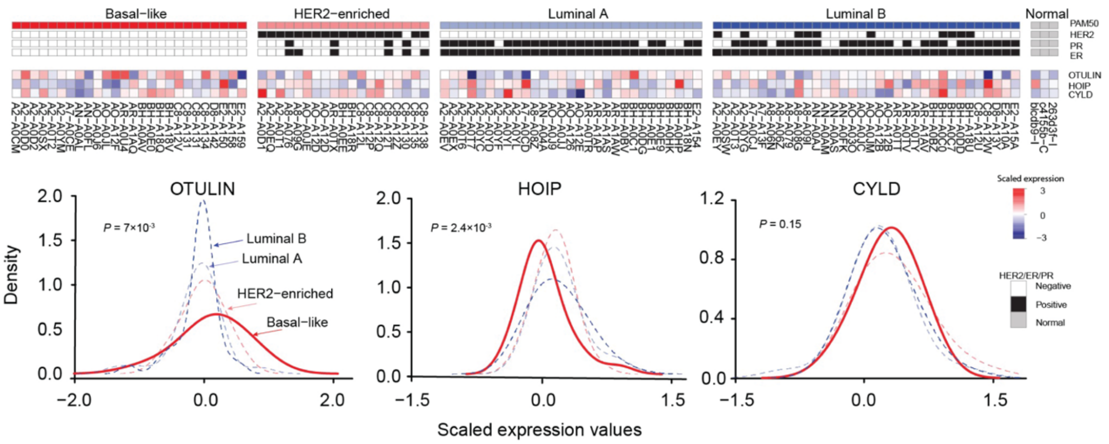

B

BLBC vs.	CYLD	OTULIN	HOIP
Others	1.03	1.16	0.90
Ctrl	1.18	1.41	0.86

Fig. S6. Bioinformatic analysis. (*A*) Expression of OTULIN, HOIP, and CYLD proteins across 111 patient samples. In the upper panels, the expression of the three proteins (i.e., OTULIN, HOIP, and CYLD) across 108 breast tumor samples and 3 normal breast tissue samples was shown in heatmap. The tumor samples were divided into four subtypes: basal-like, red (*n* = 26, biological samples); HER2-enriched, pink (*n* = 19); luminal subtype A, light blue (*n* = 29); luminal subtype B, dark blue (*n* = 34). Normalized isobaric tags for relative and absolute quantitation (iTRAQ) protein abundance ratio was shown for each protein. The expression data were downloaded from the Clinical Proteomic Tumor Analysis Consortium (CPTAC) Data Portal (https://cptac-data-portal.georgetown.edu/cptac/s/S029). The log2-transformed and then Z-score–normalized quantitative data of these three proteins were used for the analysis and plots. Distributions of expression of OTULIN, HOIP, and CYLD proteins among the four tumor types in the lower panels. $p < 0.05$ is considered significant (basal-like vs. others). BLBC, basal-like breast cancer. (*B*) Fold change of the expression of CYLD, OTULIN, and HOIP in basal-like breast cancer vs. other breast cancers and basal-like breast cancer vs. normal breast tissue.

Table S1. SgRNA sequences for cell gene knockout.

Sg1 HOIP F	5'-CACCGGTTGAGCTTCCCCGAAGGGC-3'
Sg1 HOIP R	5'-AAACGCCCTTCGGGGAAGCTCAACC-3'
Sg2 HOIP F	5'-CACCGGCACTGCCCATCCTGTAAAC-3'
Sg2 HOIP R	5'-AAACGTTTACAGGATGGGCAGTGCC-3'
Sg1 OTULIN F	5’-CACCGCGAGCGACCGCATGAGTCGG-3'
Sg1 OTULIN R	5'-AAACCCGACTCATGCGGTCGCTCGC-3'
Sg2 OTULIN F	5'-CACCGCGCGGACTCACTGCTCGGCC-3'
Sg2 OTULIN R	5'-AAACGGCCGAGCAGTGAGTCCGCGC-3'
Sg3 OTULIN F	5'-CACCGCAGCGTACCAGCATGAGCTC-3'
Sg3 OTULIN R	5'-AAACGAGCTCATGCTGGTACGCTGC-3'

Table S2. Primers for the generation of the OTULIN mutants.

OTULIN C129S F	5'-CGTGGTGATAATTACTCTGCACTGAGGGCCACG-3'
OTULIN C129S R	5'-CGTGGCCCTCAGTGCAGAGTAATTATCACCACG-3'
OTULIN Y56F F	5'-CATGAGGAGGACATGTTCCGTGCTGCAGATGA-3'
OTULIN Y56F R	5'-TCATCTGCAGCACGGAACATGTCCTCCTCATG-3'
OTULIN K34R F	5'-CACGGCGCGGGACGGCGGGAGGGCGGCGGCCAGCGGGCAG-3'
OTULIN K34R R	5'-CTGCCCGCTGGCCGCCGCCCTCCCGCCGTCCCGCGCCGTG-3'
OTULIN K64/66R F	5'-CAGATGAAATAGAAAGGGAGAGAGAATTGCTTATACA-3'
OTULIN K64/66R R	5'-TGTATAAGCAATTCTCTCTCCCTTTCTATTTCATCTG-3'
OTULIN C17A F	5'-AGGCGCGAGCGCTGCCGAGAC-3'
OTULIN C17A R	5'-GTCTCGGCAGCGCTCGCGCCT-3'
OTULIN C47A F	5'-AGATGCAGGCTCCGGCCGAGCATGAG-3'
OTULIN C47A R	5'-CTCATGCTCGGCCGGAGCCTGCATCT-3'

Dataset S1. The ubiquitination analysis of OTULIN by MS.

Dataset S2. The alkylation analysis of OTULIN by MS.

SI References

1. S. Bisen, D. Kakhniashvili, D. L. Johnson, A. N. Bukiya, Proteomic Analysis of Baboon Cerebral Artery Reveals Potential Pathways of Damage by Prenatal Alcohol Exposure. *Mol Cell Proteomics* 18, 294-307 (2019).
